# Highly perturbed genes and hub genes associated with type 2 diabetes in different tissues of adult humans: a bioinformatics analytic workflow

**DOI:** 10.1007/s10142-022-00881-5

**Published:** 2022-07-05

**Authors:** Kushan De Silva, Ryan T. Demmer, Daniel Jönsson, Aya Mousa, Andrew Forbes, Joanne Enticott

**Affiliations:** 1 Monash Centre for Health Research and Implementation, School of Public Health and Preventive Medicine, Faculty of Medicine, Nursing, and Health Sciences, Monash University, Clayton, 3168 Australia; 2 Division of Epidemiology and Community Health, School of Public Health, University of Minnesota, Minneapolis, MN USA; 3 Mailman School of Public Health, Columbia University, New York, NY USA; 4 Department of Periodontology, Faculty of Odontology, Malmö University, 21119 Malmö, Sweden; 5 Department of Clinical Sciences, Lund University, 21428 Malmö, Sweden; 6 Biostatistics Unit, Division of Research Methodology, School of Public Health and Preventive Medicine, Faculty of Medicine, Nursing, and Health Sciences, Monash University, Melbourne, 3004 Australia

**Keywords:** Differential gene expression, Highly perturbed genes, Hub genes, Meta-analysis, Type 2 diabetes

## Abstract

Type 2 diabetes (T2D) has a complex etiology which is not yet fully elucidated. The identification of gene perturbations and hub genes of T2D may deepen our understanding of its genetic basis. We aimed to identify highly perturbed genes and hub genes associated with T2D via an extensive bioinformatics analytic workflow consisting of five steps: systematic review of Gene Expression Omnibus and associated literature; identification and classification of differentially expressed genes (DEGs); identification of highly perturbed genes via meta-analysis; identification of hub genes via network analysis; and downstream analysis of highly perturbed genes and hub genes. Three meta-analytic strategies, random effects model, vote-counting approach, and *p* value combining approach, were applied. Hub genes were defined as those nodes having above-average betweenness, closeness, and degree in the network. Downstream analyses included gene ontologies, Kyoto Encyclopedia of Genes and Genomes pathways, metabolomics, COVID-19-related gene sets, and Genotype-Tissue Expression profiles. Analysis of 27 eligible microarrays identified 6284 DEGs (4592 downregulated and 1692 upregulated) in four tissue types. Tissue-specific gene expression was significantly greater than tissue non-specific (shared) gene expression. Analyses revealed 79 highly perturbed genes and 28 hub genes. Downstream analyses identified enrichments of shared genes with certain other diabetes phenotypes; insulin synthesis and action-related pathways and metabolomics; mechanistic associations with apoptosis and immunity-related pathways; COVID-19-related gene sets; and cell types demonstrating over- and under-expression of marker genes of T2D. Our approach provided valuable insights on T2D pathogenesis and pathophysiological manifestations. Broader utility of this pipeline beyond T2D is envisaged.

**Supplementary Information:**

The online version contains supplementary material available at 10.1007/s10142-022-00881-5.

## Introduction

According to an analysis of global data through years 1990–2018, diabetes was prevalent in almost half a billion people, a number expected to rise by 25% and 51%, respectively, by 2030 and 2045 (Saeedi et al. 2019). Type 2 diabetes (T2D) accounts for over 90% of all diabetes cases (Zheng et al. 2018), affecting nearly 6.8% (537 million) of the world population in year 2021 (Sun et al. 2022). Current evidence suggests a complex and multifactorial etiology of T2D characterized by genetic and environmental interactions (Arroyo et al. 2021), although T2D pathogenesis is not yet fully elucidated. There are likely varying degrees of shared genetic origins in the pathogenesis of T2D and other diabetes phenotypes such as type 1 diabetes (T1D) (Aylward et al. 2018), latent autoimmune diabetes in adults (Basile et al. 2014), and maturity-onset diabetes of the young (MODY) (Bonnefond et al. 2020). Recent studies also support the deconstruction of T2D heterogeneity to define T2D sub-types (Udler et al. 2018) and the delineation of a continuum of diabetes sub-types (Flannick et al. 2016) instead of the status quo characterized by a few distinct diabetes phenotypes. Understanding the genetic basis of T2D is fundamental to precision medicine approaches striving for impeccable matching and an individualized level of T2D care (Prasad and Groop 2019).

Cardinal tissues of the body impacted by heightened insulin resistance and diminished insulin secretion in T2D include the pancreas, liver, skeletal muscle, and adipose tissue (Batista et al. 2021). Exploration of gene perturbations in these tissues can deepen our understanding of the molecular etio-pathology of T2D. Highly up- and downregulated genes expressed consistently across different tissue types may uncover potential genome-wide biomarkers or “gene signatures,” which are integral to achieving precision diagnostic, prognostic, monitoring, and treatment approaches. Topologically, hub genes are defined as highly and tightly connected nodes in typically scale-free gene regulatory networks (GRN) (Buchberger et al. 2021). As such, criteria such as high correlation in candidate modules (Liu et al. 2019a) and above-average betweenness, closeness, and degree (Liu et al. 2019b) in GRN have been used to demarcate hub genes in previous studies. Functionally, they perform critical regulatory roles in biological processes interacting with many other genes in associated pathways. Given their crucial structural and functional characteristics, hub genes are highly sought-after in precision medicine approaches as plausible niches for developing drug and treatment targets (Liu et al. 2021). In this context, the importance of identifying highly perturbed genes and hub genes associated with T2D for the purpose of individualizing T2D care is unequivocal.

Downstream analyses of gene sets provide invaluable insights into associated core biological functions, pathways, diseases, drugs, and many other aspects. Frequently used gene ontology (GO) analysis provides evidence as a snapshot of contemporary biological knowledge related to a given gene including its function at the molecular level, the cellular location(s) it functions at, and the biological processes reliant on it (Hill et al. 2008). Pathway analyses such as Kyoto Encyclopedia of Genes and Genomes (KEGG) (Kanehisa and Goto 2000) entail derivation of coherent and meaningful biological phenomena attributable to input genes (Nguyen et al. 2019). Additional downstream analyses of diseases, drugs, metabolomes, and tissue enrichment are also available and can provide valuable insights into associated genes. Taken together, downstream analysis of highly perturbed and hub genes associated with T2D may render valuable information on aspects such as affected biological processes, dysregulated pathways, related diseases, and metabolomic biomarkers.

Advances in high-throughput technologies have generated a wealth of gene expression data, while the availability of open-source platforms such as the National Center for Biotechnology Information Gene Expression Omnibus (NCBI GEO) (Edgar et al. 2002; Barrett et al. 2013) and simultaneous advent in big data and bioinformatics analytic tools such as microarray and RNA-seq meta-analysis and gene–gene interaction network analysis strategies have offered unprecedented opportunities for high-level evidence synthesis from a multitude of gene expression datasets. Such approaches are likely to render new knowledge on complex diseases like T2D and acquire adequate statistical power to identify genes associated with a disease that may not have been evoked via prior analysis of a single or a few datasets.

Yet, to date, no comprehensive evidence synthesis study has been performed to identify highly perturbed genes and hub genes associated with T2D in human adults using an extensive bioinformatics analytic pipeline. Prior studies were limited to identifying hub genes in a few (*n* = 3) microarrays from a single tissue type (pancreatic islets) (Lin et al. 2020) or performing an ad hoc gene expression meta-analysis of all diabetes phenotypes (*n* = 13) (Mei et al. 2017). In this study, we aimed to identify highly perturbed genes and hub genes associated with T2D in different tissues of adult humans via a pre-defined and extensive bioinformatics analytic workflow consisting of systematic review, meta-analysis, identification and classification of differentially expressed genes (DEGs), network analysis, and downstream analysis.

## Methods

The methodological approach consisted of five sequential steps: (1) systematic review of NCBI GEO expression data and related publications, (2) analysis of microarrays to identify DEGs, (3) meta-analysis of DEGs to identify highly perturbed genes in T2D, (4) network analysis of gene–gene/protein–protein interactions to identify hub genes in T2D, and (5) downstream analysis of highly perturbed genes and hub genes associated with T2D.

### Systematic review

A preliminary search on the NCBI GEO database was first run on 1st February 2021 using a pre-defined search string: “(“diabetes mellitus, type 2” [MeSH Terms] OR type 2 diabetes[All Fields]) AND “Homo sapiens” [porgn] AND “Expression profiling by array” [Filter].” The resulting microarrays and related publications were further screened against pre-defined eligibility criteria. Microarrays of conditions other than T2D, all diabetes phenotypes other than T2D, and early dysglycemic conditions such as impaired glucose tolerance, insulin resistance, and prediabetes were excluded. Studies with non-human specimens, children, without healthy controls, or with notable comorbidity in control samples were also excluded. We also omitted studies involving long non-coding RNA (lncRNA), micro RNA (miRNA), samples subject to drug treatments and other interventions, pluripotent stem cells, xenografts, transfected or transgenic tissues, undifferentiated tissues, and sub-samples in super-series. Microarrays passing these eligibility criteria were selected for manual curation which were then further screened along with the full texts of related publications (where available) for the presence of adequate information such as clinical diagnosis (healthy vs T2D) and gene symbol/Entrez ID. Following this, all microarrays with sufficient information were selected for subsequent analyses.

### Identification of DEGs

All microarrays selected from the systematic review were imported to *R* using *getGEO* function of *GEOquery* package (Davis and Meltzer 2007). In each expression set, *phenoData* component was examined to determine the number of eligible samples and confirm the presence of outcome (T2D vs controls) variable, while *featureData* component was explored to verify the presence of gene annotation information. Where required, non-normalized gene expression matrices were log_2_ transformed in order to alleviate skewness and create symmetric distributions (Le et al. 2020). Samples were assessed for the presence of any batch effects between the two groups by running principal components analysis on transposed expression matrices and were rectified using *removeBatchEffect* function in *limma* package (Ritchie et al. 2015). Relevant features were annotated with expression matrices to generate curated data for running differential gene expression analysis. Samples were ascribed to the relevant group (T2D/control) using *model.matrix* function of *limma* package (Ritchie et al. 2015) producing a binary design matrix. As the detection of DEGs can be enhanced by filtering genes with a low expression level, we assumed a median (50%) cut-off for the gene expression level. A uniform analytic pipeline consisting of the following sequential steps was applied to each microarray: (1) median expression levels were calculated, and those above the median were retained. (2) From the resulting genes, those expressed in more than two samples were retained, while the others were removed. (3) Model fitting was performed using *lmFit* function of *limma* (Ritchie et al. 2015) to enumerate expression levels of T2D and control groups. (4) Contrasts were defined as “T2D, control,” and *empirical Bayes* step was run to derive differential expression results. (5) The DEGs, defined as those with log_2_FC > 1 and Benjamini–Hochberg (BH)-adjusted *p* < 0.05 for upregulated genes and log_2_FC <  − 0.5 and BH-adjusted *p* < 0.05 for downregulated genes, were identified for each microarray.

Microarrays with no DEGs were excluded, and those with non-zero DEGs were visualized with a clustered bar chart. Furthermore, DEGs were classified by tissue types and visualized as a Venn diagram. Information on clinical and other features of the microarrays with non-zero DEGs, individual DEGs identified by each dataset, and tissue-based classification of DEGs were also summarized.

### Meta-analysis of DEGs: identification of highly perturbed genes

In order to identify highly perturbed genes associated with T2D, we conducted meta-analysis of DEGs using *MetaVolcanoR* package (Prada et al. 2020). We implemented all 3 meta-analytic strategies incorporated in this package, namely, random effects model (REM), vote-counting approach (VC), and *p* value combining approach (CA).

In brief, REM synthesizes a summary fold-change of multiple microarrays based on variance, producing a summary *p* value which indicates the probability that the summary fold-change is not different to zero. The *metathr* parameter can be specified to filter the desired percentage of the top-most consistently perturbed genes. Gene perturbation is ranked as per the *topconfects* approach (Harrison et al. 2019). The VC algorithm produces highly perturbed genes according to user-specified *p* values and fold-change cut-off levels, taking into account both the number of studies in which a DEG appeared and its gene fold-change sign consistency. Here also, *metathr* parameter can be defined to extract the required percentage of highly perturbed genes. Meta-synthesis of gene perturbation by CA algorithm is at the mean or median level along with *p* values derived by Fisher method. A required proportion of top-most DEGs can be identified by specifying *metathr* parameter with CA as well (Prada et al. 2020).

As required by the package, all microarray datasets with non-zero DEGs; each consisting of the columns gene name (symbol), fold-change (log_2_FC), and *p* value; and confidence intervals of the fold-change (CI.L and CI.R) were merged to build a list item. For all 3 meta-analytic models, *metathr* was set at 0.01. For VC, *p* value and absolute fold-change thresholds were set at 0.05 and 0, respectively.

Highly perturbed genes identified by each model as well as the compiled list of all highly perturbed genes were presented in tabular format. Volcano plots were drawn to illustrate the top-most perturbed genes identified by REM, VC, and CA methods. Inverse cumulative distribution of consistently differentially expressed genes as per VC was plotted to demonstrate the number of genes with perturbed expression in ≥ 1 studies. Detailed meta-analytic outputs from all 3 approaches and the highly perturbed genes identified by each method were also compiled.

### Network analysis: identification of hub genes

The list of highly perturbed genes was fed into GENEMANIA (Warde-Farley et al. 2010) to determine the gene–gene interaction network. The interaction network formulated by the GENEMANIA gene function prediction program, based on the multiple association network integration algorithm (MANIA), incorporates a multitude of functional associations including co-expression, pathways, physical interactions, co-localization, genetic interactions, and protein domain similarity. It has been found more accurate and computationally efficient than other gene function prediction methods (Mostafavi et al. 2008; Peña-Castillo et al. 2008). The gene–gene interaction network was first constructed and visualized in GENEMANIA. Next, these interactions were imported to visualize the protein–protein interaction (PPI) network in STRING version 11.0 (Szklarczyk et al. 2019). We used the *Centiscape* application (Scardoni et al. 2009) in the *Cytoscape* software (Shannon et al. 2003) to analyze the PPI network and determine the hub nodes. After removing nodes with a connection number < 2, the network was visualized in *Cytoscape*. Hub genes were defined as those nodes in the network with betweenness, closeness, and degree higher than their mean values. A similar approach has been previously used to demarcate hub genes (Liu et al. 2019b). Topological features of the hub nodes and details of the PPI network derived by *Centiscape* were summarized.

### Downstream analysis of highly perturbed genes and hub genes

Using *Enrichr* (Chen et al. 2013; Kuleshov et al. 2016; Xie et al. 2021) platform, we ran a series of downstream analyses for both highly perturbed genes and hub genes as outlined below:

- Ontologies: GO Biological Process 2018 (Hill et al. 2008)
- Pathways: KEGG 2019 Human (Kanehisa and Goto 2000)
- Diseases/drugs: COVID-19-related gene sets
- Cell types: GTEx tissue sample gene expression profiles up and GTEx tissue sample gene expression profiles down
- Miscellaneous: HMDB metabolites

The Genotype-Tissue Expression (GTEx) portal contains tissue-specific gene expression and regulation data (GTEx Consortium 2013), whereas the Human Metabolome Database (HMDB) records human metabolomics data (Wishart et al. 2018).

## Results

The bioinformatic analytic workflow is summarized in Online Resource 1.

### Systematic review outputs

The preliminary search resulted in 178 eligible microarrays, while 45 of these were selected for manual curation. We selected 27 microarrays with sufficient information for subsequent analyses, the details of which are provided in Online Resource 2.

### Differential gene expression analysis outputs

**Fig. 1:**
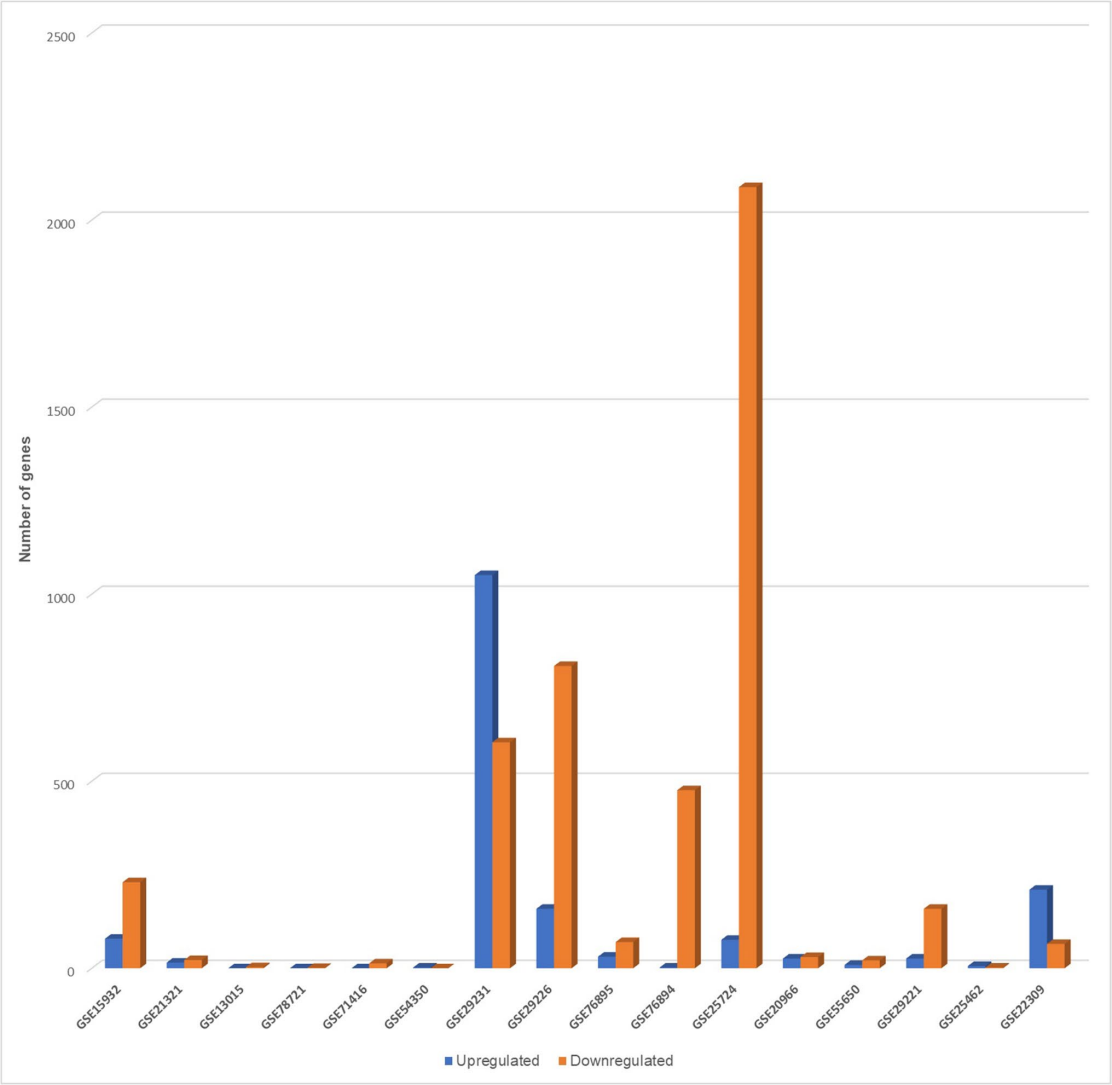
Clustered bar chart depicting the number of differentially expressed (up- and downregulated) genes in the 16 datasets

**Fig. 2:**
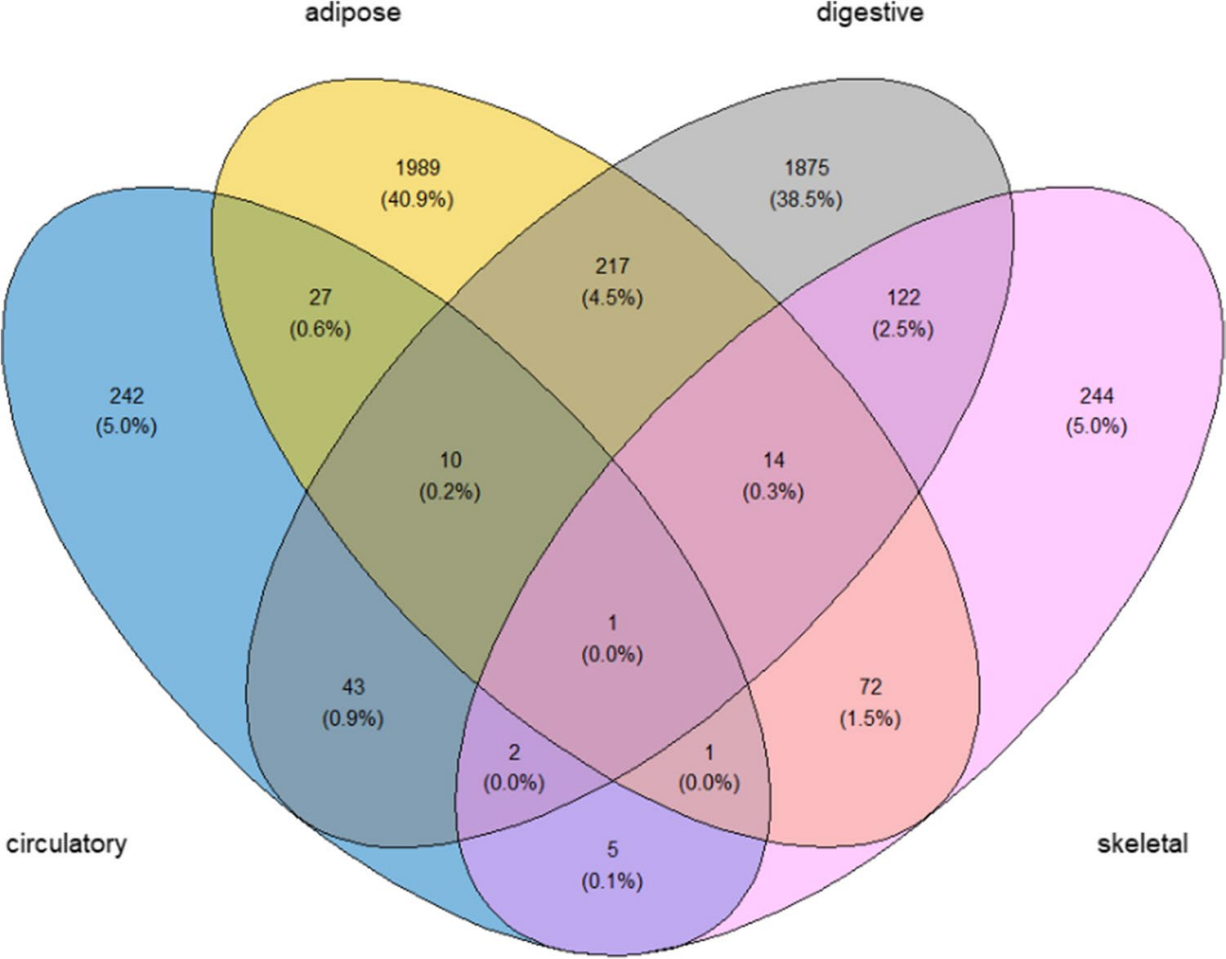
Venn diagram depicting the number of differentially expressed genes by the 4 tissue types

The number of DEGs identified by each dataset is shown in Table 1. There were 11 microarrays with no DEGs. In Fig. 1, microarrays with non-zero DEGs (*n* = 16) are visualized as a clustered bar chart. The identified DEGs belonged to four tissue types (i.e., circulatory; adipose; digestive; skeletal muscle) as visualized in the Venn diagram (Fig. 2). Clinical and other information of the 16 microarrays with non-zero DEGs is presented in Online Resource 3. Details of DEGs identified by each dataset are presented in Online Resource 4, while DEGs classified by tissue type are presented in Online Resource 5.

**Table 1:** Number of differentially expressed genes in type 2 diabetes identified from different tissues of adult humans (*n* = 27)

Dataset	Number of DEGs^a^ (*n* = 6284)	Upregulated^b^ (*n* = 1692)	Downregulated^c^ (*n* = 4592)	Tissue
GSE156993	0	0	0	PBMCs
GSE15932	309	79	230	Peripheral blood
GSE21321	37	15	22	Peripheral blood
GSE13015	3	0	3	Whole blood
GSE13760	0	0	0	Arterial tissue
GSE78721	1	0	1	Adipocytes
GSE71416	13	0	13	Omental adipose tissue
GSE54350	2	2	0	Visceral adipose tissue
GSE29231	1655	1051	604	Visceral adipose tissue
GSE16415	0	0	0	Visceral adipose tissue
GSE29226	967	159	808	Subcutaneous adipose tissue
GSE27949	0	0	0	Subcutaneous adipose tissue
GSE76895	101	31	70	Pancreatic islets
GSE76894	478	2	476	Pancreatic islets
GSE38642	0	0	0	Pancreatic islets
GSE25724	2164	76	2088	Pancreatic islets
GSE20966	56	26	30	Beta cells from pancreatic tissue
GSE64998	0	0	0	Liver
GSE23343	0	0	0	Liver
GSE15653	0	0	0	Liver
GSE73034	0	0	0	Skeletal muscle
GSE55650	30	9	21	Skeletal muscle
GSE29221	185	26	159	Skeletal muscle
GSE25462	8	6	2	Skeletal muscle
GSE19420	0	0	0	Skeletal muscle
GSE22309	275	210	65	Skeletal muscle
GSE21340	0	0	0	Skeletal muscle

a, differentially expressed genes were defined as BH-adjusted *p* value < 0.05 and log_2_FC > 1|log_2_FC <  − 0.5; b, upregulated genes were defined as BH-adjusted *p* value < 0.05 and log_2_FC > 1; c, downregulated genes were defined as BH-adjusted *p* value < 0.05 and log_2_FC <  − 0.5

*DEGs*, differentially expressed genes; *PBMCs*, peripheral blood mononuclear cells

### A significantly larger proportion of genes associated with T2D is downregulated

Of all the DEGs identified by different tissues (*n* = 6284), the proportion of downregulated genes (*n* = 4592) was significantly higher than the proportion of upregulated genes (*n* = 1692) (*p* < 0.00000001). At the level of tissue type, circulatory (255 downregulated vs 94 upregulated; *p* < 0.000001), adipose (1426 downregulated vs 1212 upregulated; *p* = 0.000017), and digestive (2664 downregulated vs 135 upregulated; *p* < 0.000001) tissues revealed a similar pattern, while the skeletal muscle (247 downregulated vs 251 upregulated; *p* = 0.44653) showed no such over-expression of downregulated genes. Of the 27 microarrays, 11 contained no DEGs, while the two datasets containing the highest number of DEGs were from pancreatic islets (GSE25724; 2164 DEGs) and visceral adipose tissue (GSE29231; 1655 DEGs) (Table 1, Fig. 1).

### Tissue-specific DEGs are predominant compared to DEGs shared between tissues in T2D

Proportions of tissue-specific DEGs were significantly higher than the proportions shared with one or more other tissue types in circulatory (242 specific vs 89 non-specific; *p* < 0.000001), adipose (1989 specific vs 342 non-specific; *p* < 0.000001), and digestive (1875 specific vs 409 non-specific; *p* < 0.000001) groups, while skeletal muscle (244 specific vs 217 non-specific; *p* = 0.112959) had no such over-expression (Fig. 2).

### Meta-analytic outputs

**Fig. 3:**
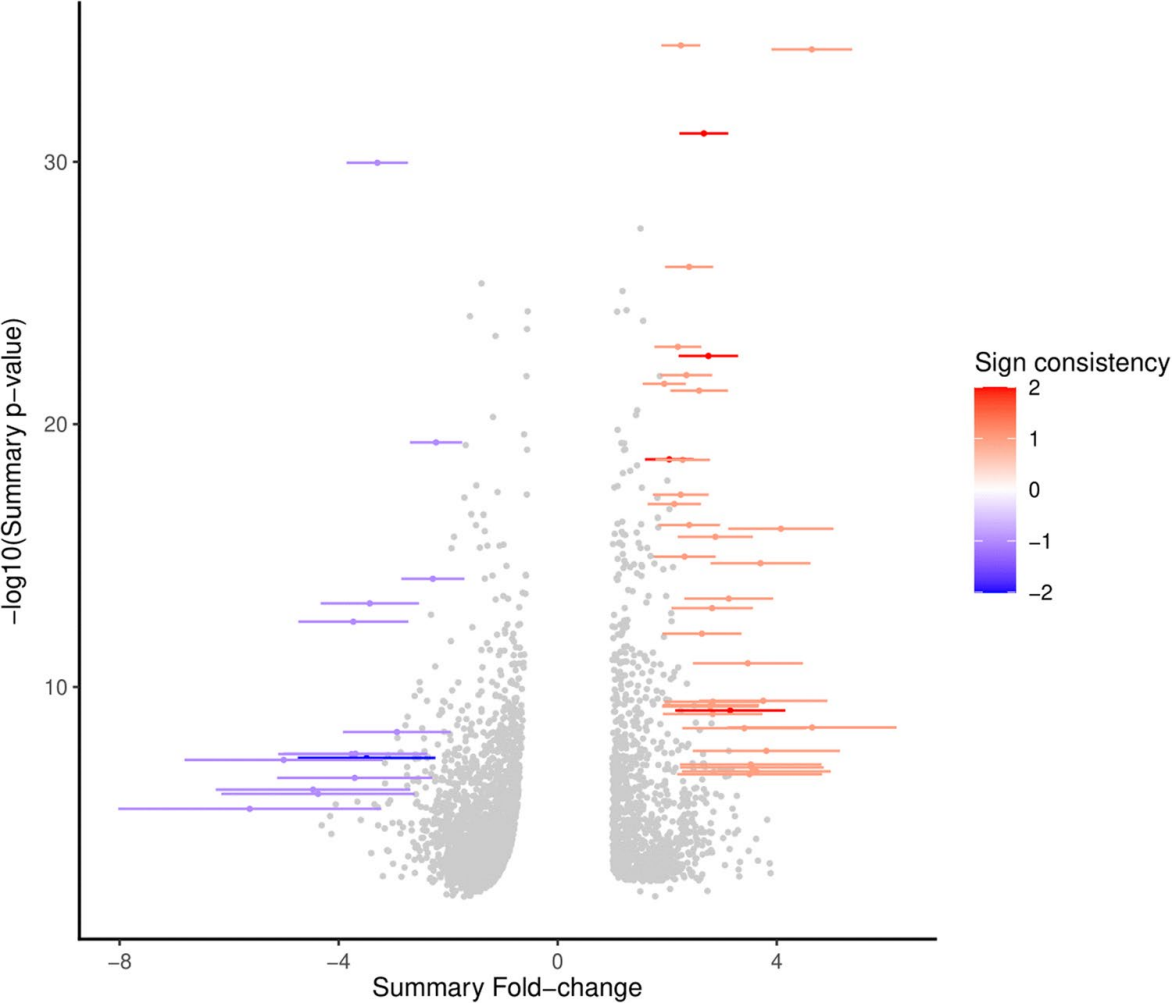
Highly perturbed genes (*n* = 49) identified by random effects model meta-analysis in *MetaVolcanoR* package with *metathr* set at 0.01. Consistently upregulated genes appear in red and consistently downregulated genes appear in blue

**Fig. 4:**
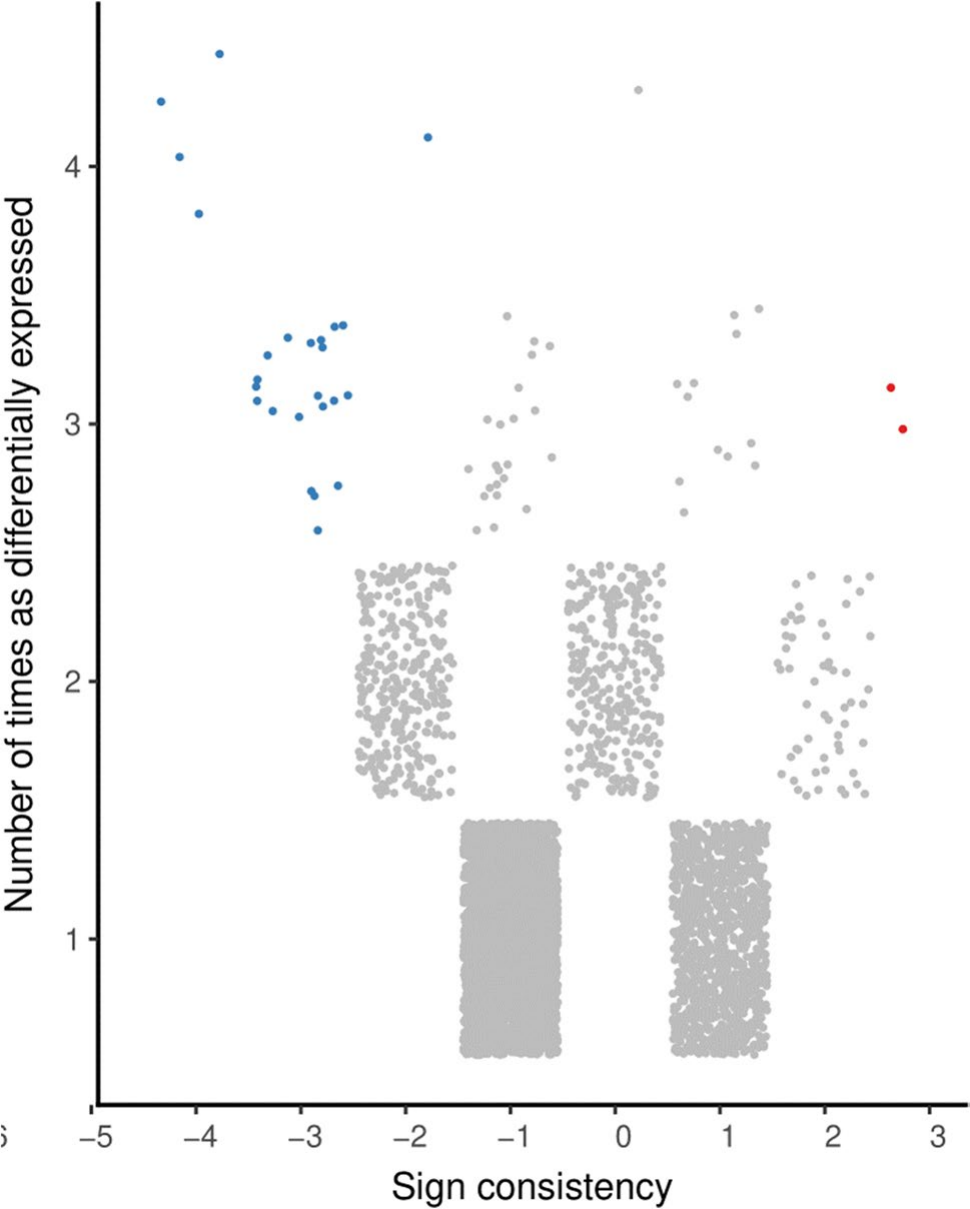
Highly perturbed genes (*n* = 27) identified by vote-counting approach meta-analysis in *MetaVolcanoR* package with *metathr* set at 0.01. Consistently upregulated genes appear in red and consistently downregulated genes appear in blue

**Fig. 5:**
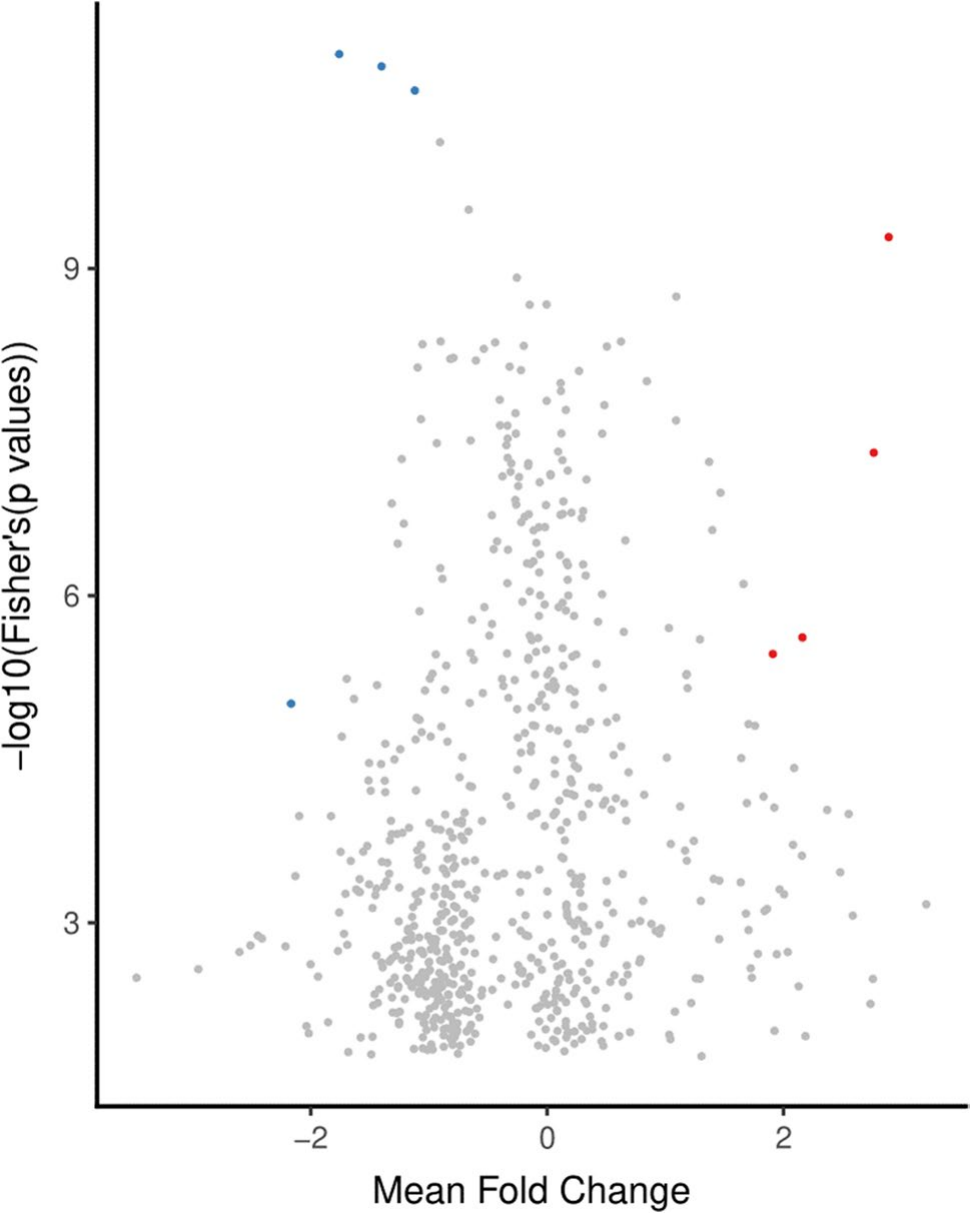
Highly perturbed genes (*n* = 8) identified by *p* value combining approach meta-analysis in *MetaVolcanoR* package with *metathr* set at 0.01. Consistently upregulated genes appear in red and consistently downregulated genes appear in blue

**Fig. 6:**
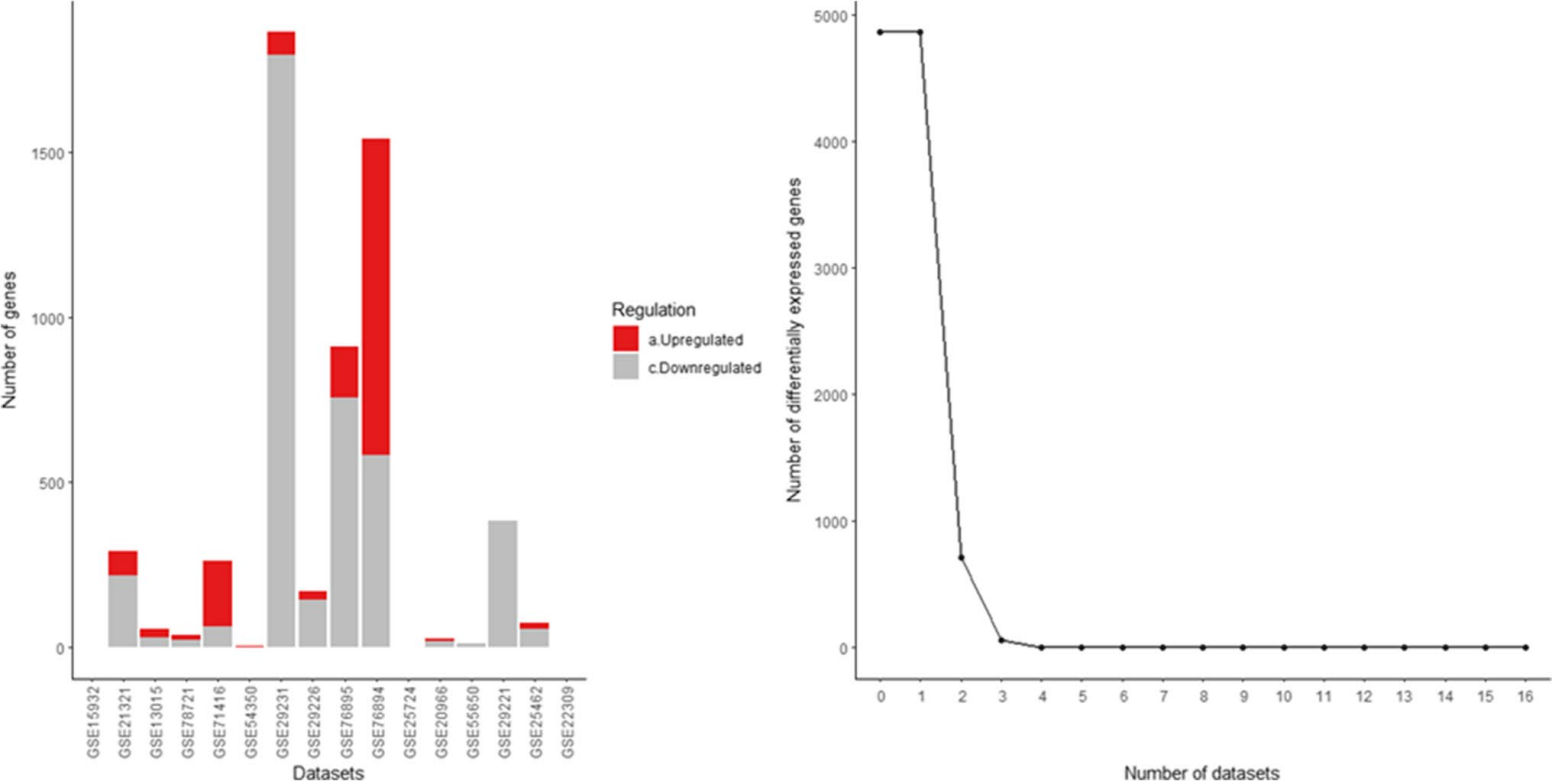
Inverse cumulative distribution of the consistently differentially expressed genes as per vote-counting approach

As shown in Table 2, the three meta-analytic algorithms REM, VC, and CA identified 49, 27, and 8 highly perturbed genes, respectively. The compiled list, after removing redundancies, comprised 79 highly perturbed genes. Volcano plots illustrating the top-most perturbed genes identified by REM, VC, and CA are given in Figs. 3, 4, 5, respectively. Figure 6 presents the inverse cumulative distribution of consistently differentially expressed genes as per VC, plotted to demonstrate the number of genes with perturbed expression in ≥ 1 studies. We present detailed meta-analytic outputs from all 3 approaches in Online Resource 6 and highly perturbed genes identified by each method in Online Resource 7.

**Table 2:** Highly perturbed genes (*n* = 79) associated with type 2 diabetes identified by meta-analysis of microarray (*n* = 16)

Approach	Highly perturbed genes	Upregulated genes	Downregulated genes
Random effects model	49 genes: *XYLT1, ISLR, CRTAC1, ERAP2, PCOLCE2, VNN2, U2AF2, NPTX2, MMP9, LOC100008589, DYRK3, LOC649456, BCL3, ZNF423, SOD3, CNTFR, ZNF75, RNF19B, TNFAIP6, TAPBP, PHACTR3, PHLDA1, ALDOB, PRIMA1, PVRL2, CNTNAP2, RASL11B, POMZP3, ELFN1, ESPNL, PI3, SCN1B, EGR2, MGRN1, SLC9A3R2, LOC650885, UGT2B7, C19orf33, HLA-DRB4, OR8B12, HLA-DRB5, IFNA7, LOC389286, FUT11, APOL4, LOC731682, KIAA1984, POPDC3, TAP2*	35 genes: *XYLT1, ISLR, CRTAC1, ERAP2, PCOLCE2, VNN2, U2AF2, NPTX2, MMP9, LOC100008589, DYRK3, LOC649456, BCL3, ZNF423, SOD3, CNTFR, ZNF75, RNF19B, TNFAIP6, TAPBP, PHACTR3, PHLDA1, ALDOB, PRIMA1, PVRL2, CNTNAP2, RASL11B, POMZP3, ELFN1, ESPNL, PI3, SCN1B, EGR2, MGRN1, SLC9A3R2*	14 genes: *LOC650885, UGT2B7, C19orf33, HLA-DRB4, OR8B12, HLA-DRB5, IFNA7, LOC389286, FUT11, APOL4, LOC731682, KIAA1984, POPDC3, TAP2*
Vote-counting approach	27 genes: *LOC644422, MCL1, CTSC, DYRK2, MCOLN3, TMEM37, API5, ARG2, C14orf132, COG2, DHRS2, ENPP2, ENTPD3, HADH, KIAA1279, LARP4, MARK1, MTRR, NAALAD2, PAAF1, PPM1K, PPP1R1A, RPL14, SLC2A2, SNAP25, STMN2, NMNAT2*	2 genes: *LOC644422, MCL1*	25 genes: *CTSC, DYRK2, MCOLN3, TMEM37, API5, ARG2, C14orf132, COG2, DHRS2, ENPP2, ENTPD3, HADH, KIAA1279, LARP4, MARK1, MTRR, NAALAD2, PAAF1, PPM1K, PPP1R1A, RPL14, SLC2A2, SNAP25, STMN2, NMNAT2*
Combining approach	8 genes: *PCOLCE2, ERAP2, EGR2, ZBTB16, SLC2A2, ARG2, ASCL2, IAPP*	4 genes: *PCOLCE2, ERAP2, EGR2, ZBTB16*	4 genes: *SLC2A2, ARG2, ASCL2, IAPP*
Compiled set	79 genes: *XYLT1, ISLR, CRTAC1, ERAP2, PCOLCE2, VNN2, U2AF2, NPTX2, MMP9, LOC100008589, DYRK3, LOC649456, BCL3, ZNF423, SOD3, CNTFR, ZNF75, RNF19B, TNFAIP6, TAPBP, PHACTR3, PHLDA1, ALDOB, PRIMA1, PVRL2, CNTNAP2, RASL11B, POMZP3, ELFN1, ESPNL, PI3, SCN1B, EGR2, MGRN1, SLC9A3R2, LOC644422, MCL1, ZBTB16, LOC650885, UGT2B7, C19orf33, HLA-DRB4, OR8B12, HLA-DRB5, IFNA7, LOC389286, FUT11, APOL4, LOC731682, KIAA1984, POPDC3, TAP2, CTSC, DYRK2, MCOLN3, TMEM37, API5, ARG2, C14orf132, COG2, DHRS2, ENPP2, ENTPD3, HADH, KIAA1279, LARP4, MARK1, MTRR, NAALAD2, PAAF1, PPM1K, PPP1R1A, RPL14, SLC2A2, SNAP25, STMN2, NMNAT2, ASCL2, IAPP*	38 genes: *XYLT1, ISLR, CRTAC1, ERAP2, PCOLCE2, VNN2, U2AF2, NPTX2, MMP9, LOC100008589, DYRK3, LOC649456, BCL3, ZNF423, SOD3, CNTFR, ZNF75, RNF19B, TNFAIP6, TAPBP, PHACTR3, PHLDA1, ALDOB, PRIMA1, PVRL2, CNTNAP2, RASL11B, POMZP3, ELFN1, ESPNL, PI3, SCN1B, EGR2, MGRN1, SLC9A3R2, LOC644422, MCL1, ZBTB16*	41 genes: *LOC650885, UGT2B7, C19orf33, HLA-DRB4, OR8B12, HLA-DRB5, IFNA7, LOC389286, FUT11, APOL4, LOC731682, KIAA1984, POPDC3, TAP2, CTSC, DYRK2, MCOLN3, TMEM37, API5, ARG2, C14orf132, COG2, DHRS2, ENPP2, ENTPD3, HADH, KIAA1279, LARP4, MARK1, MTRR, NAALAD2, PAAF1, PPM1K, PPP1R1A, RPL14, SLC2A2, SNAP25, STMN2, NMNAT2, ASCL2, IAPP*

### Highly perturbed genes associated with T2D comprise both up- and downregulated genes

There was no significant difference (*p* = 0.410983) between the proportions of up- (38/79) and downregulated (41/79) genes that constituted the highly perturbed gene set (*n* = 79) identified by all 3 meta-analytic algorithms (Table 2, Figs. 3, 4, 5, 6). The 38 upregulated genes were *ALDOB*; *BCL3*; *CNTFR*; *CNTNAP2*; *CRTAC1*; *DYRK3*; *EGR2*; *ELFN1*; *ERAP2*; *ESPNL*; *ISLR*; *LOC100008589*; *LOC644422*; *LOC649456*; *MCL1*; *MGRN1*; *MMP9*; *NPTX2*; *PCOLCE2*; *PHACTR3*; *PHLDA1*; *PI3*; *POMZP3*; *PRIMA1*; *PVRL2*; *RASL11B*; *RNF19B*; *SCN1B*; *SLC9A3R2*; *SOD3*; *TAPBP*; *TNFAIP6*; *U2AF2*; *VNN2*; *XYLT1*; *ZBTB16*; *ZNF423*; and *ZNF75*. The 41 downregulated genes were *API5*; *APOL4*; *ARG2*; *ASCL2*; *C14orf132*; *C19orf33*; *COG2*; *CTSC*; *DHRS2*; *DYRK2*; *ENPP2*; *ENTPD3*; *FUT11*; *HADH*; *HLA-DRB4*; *HLA-DRB5*; *IAPP*; *IFNA7*; *KIAA1279*; *KIAA1984*; *LARP4*; *LOC389286*; *LOC650885*; *LOC731682*; *MARK1*; *MCOLN3*; *MTRR*; *NAALAD2*; *NMNAT2*; *OR8B12*; *PAAF1*; *POPDC3*; *PPM1K*; *PPP1R1A*; *RPL14*; *SLC2A2*; *SNAP25*; *STMN2*; *TAP2*; *TMEM37*; and *UGT2B7*.

### Network analysis outputs

**Fig. 7:**
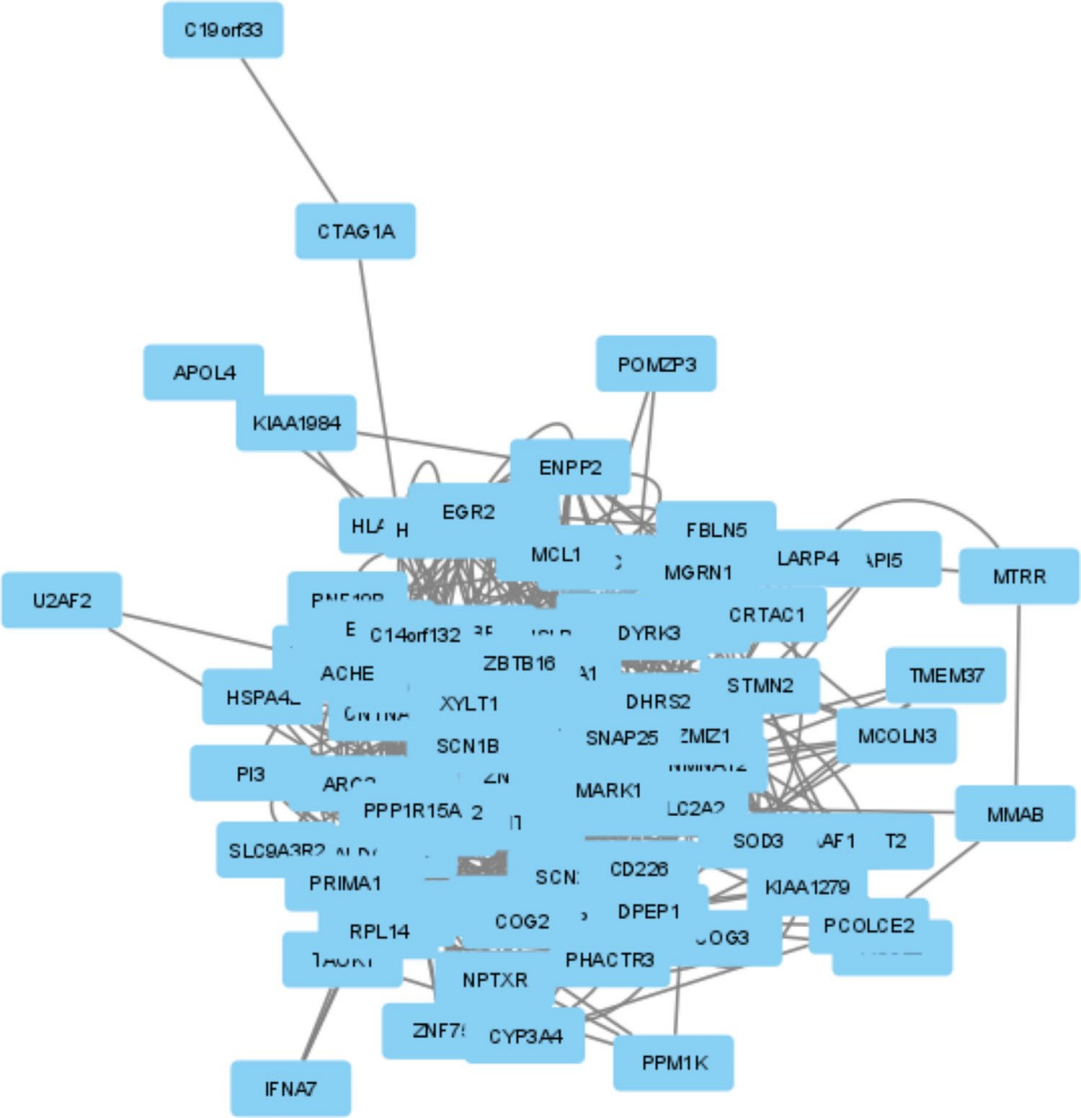
Gene–gene interactions network visualized in GENEMANIA

**Fig. 8:**
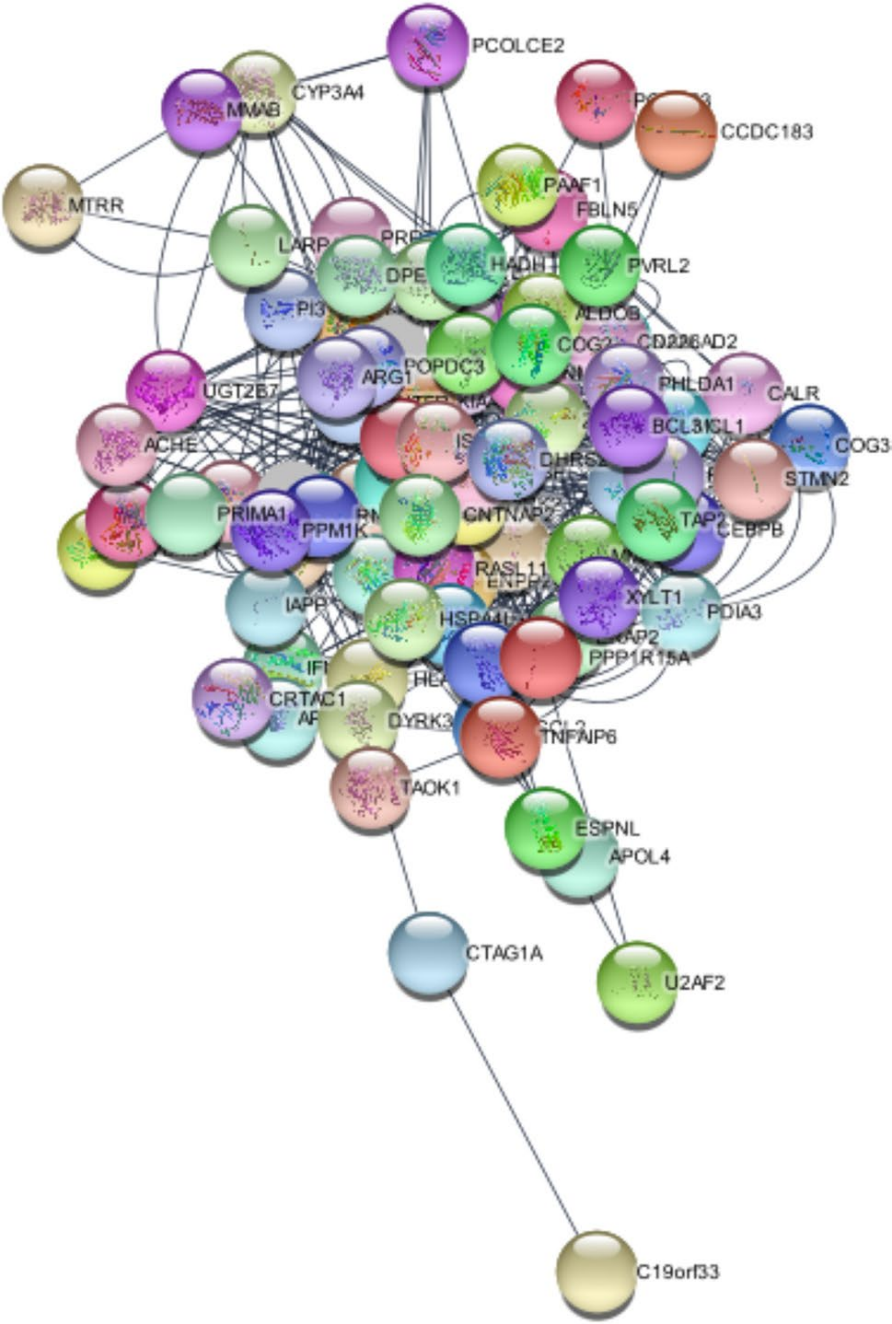
Protein–protein interactions network visualized in STRING

**Fig. 9:**
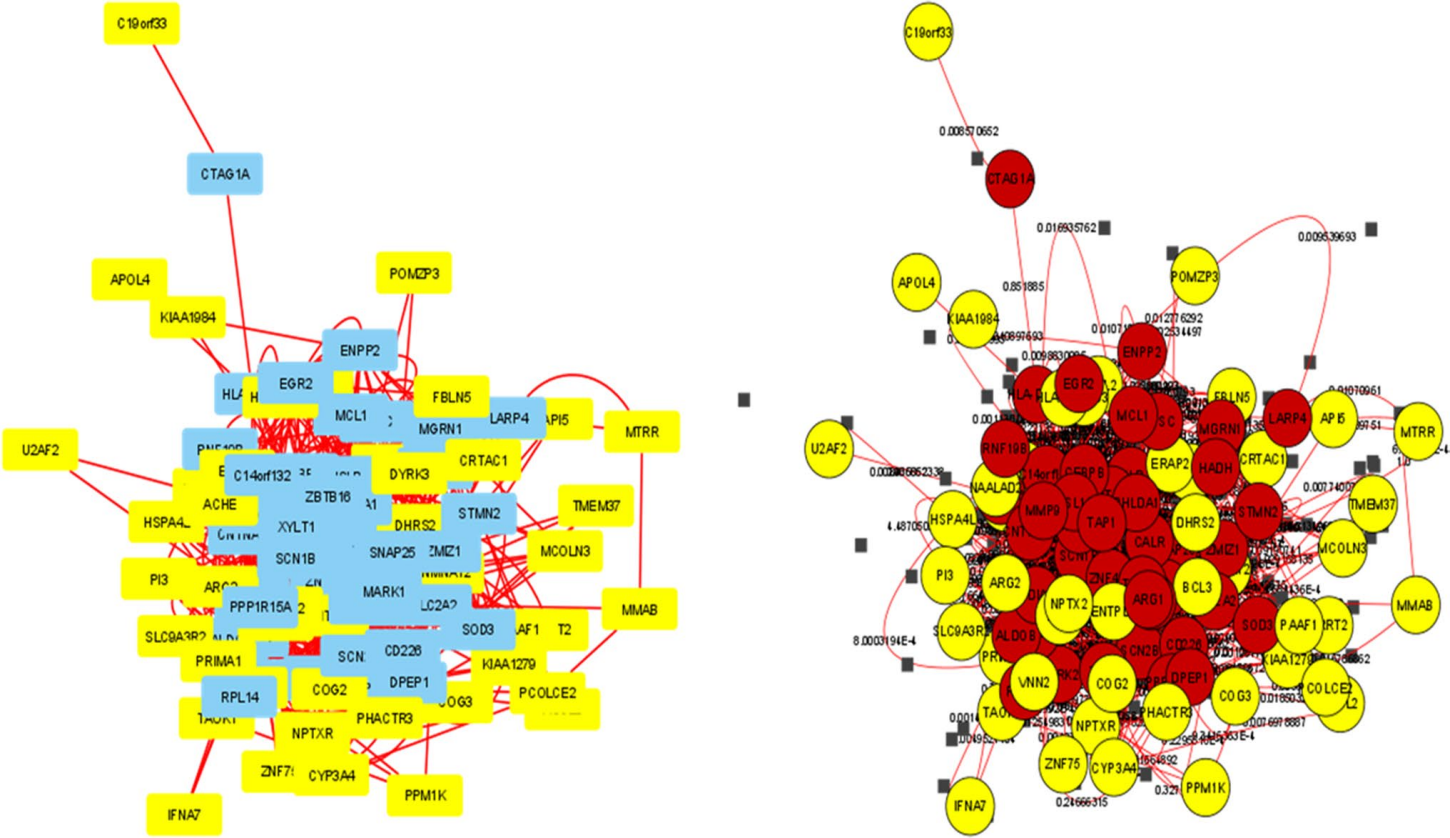
Two different presentations of the protein–protein interactions network in *Centiscape*

Network analysis identified 28 hub genes, the topological features of which are summarized in Table 3. Figure 7 presents the gene–gene interaction network produced on GENEMANIA, and details of this network are given in Online Resource 8. The PPI network visualized on STRING version 11.0 (Szklarczyk et al. 2019) is given in Fig. 8. The network created by Cytoscape is provided in Fig. 9. Details of the PPI network derived by Centiscape are provided in Online Resource 9.

**Table 3:** Topological characteristics of the hub genes (*n* = 28) identified via network analyses

Gene	Betweenness	Closeness	Degree	Regulation
	**Mean = 115.5333333**	**Mean = 0.004988249**	**Mean = 12.68888889**	
*PHLDA1*	213.0074	0.005618	22	Upregulated
*CTSC*	149.7385	0.005495	18	Downregulated
*HADH*	208.3918	0.00578	18	Downregulated
*RPL14*	139.3092	0.005464	17	Downregulated
*ZBTB16*	127.1864	0.005405	14	Upregulated
*SOD3*	198.8821	0.005714	21	Upregulated
*SNAP25*	380.1873	0.005814	30	Downregulated
*SLC2A2*	438.8331	0.005747	26	Downregulated
*ISLR*	465.6411	0.005952	27	Upregulated
*SCN1B*	175.7841	0.005376	14	Upregulated
*MMP9*	196.9649	0.005682	22	Upregulated
*ENPP2*	230.6537	0.005618	17	Downregulated
TAP1*	189.4171	0.005376	40	-
*MCL1*	320.3027	0.005917	27	Upregulated
DPEP1*	133.0483	0.005208	15	-
*CNTNAP2*	761.7546	0.006494	39	Upregulated
*STMN2*	200.277	0.005618	18	Downregulated
*PPP1R1A*	219.8601	0.005587	18	Downregulated
*ZNF423*	406.5439	0.006061	21	Upregulated
*CNTFR*	200.657	0.005319	16	Upregulated
*TNFAIP6*	162.3969	0.005208	14	Upregulated
PPP1R15A*	119.5084	0.005155	13	-
*XYLT1*	194.4998	0.00565	18	Upregulated
ZMIZ1*	392.1699	0.005682	26	-
*RASL11B*	168.9444	0.005376	13	Upregulated
ARG1*	124.961	0.005263	18	-
*UGT2B7*	247.0102	0.005682	19	Downregulated
CD226*	222.4953	0.005495	17	-

^*^Genes included in the network as predicted by GENEMANIA

### Hub genes associated with T2D also comprise both up- and downregulated genes

The 28 hub genes consisted of 13 upregulated, 9 downregulated, and 6 predicted genes. There was no significant difference (*p* = 0.261216) between the proportions of up- (13/22) and downregulated (9/22) genes constituting the hub gene set. The 13 upregulated hub genes were *CNTFR*; *CNTNAP2*; *ISLR*; *MCL1*; *MMP9*; *PHLDA1*; *RASL11B*; *SCN1B*; *SOD3*; *TNFAIP6*; *XYLT1*; *ZBTB16*; and *ZNF423*. The 9 downregulated genes were *CTSC*; *ENPP2*; *HADH*; *PPP1R1A*; *RPL14*; *SLC2A2*; *SNAP25*; *STMN2*; and *UGT2B7*. The 6 hub genes predicted by GENEMANIA were *ARG1*; *CD226*; *DPEP1*; *PPP1R15A*; *TAP1*; and *ZMIZ1* (Table 3).

### Downstream functional analysis outputs

**Fig. 10:**
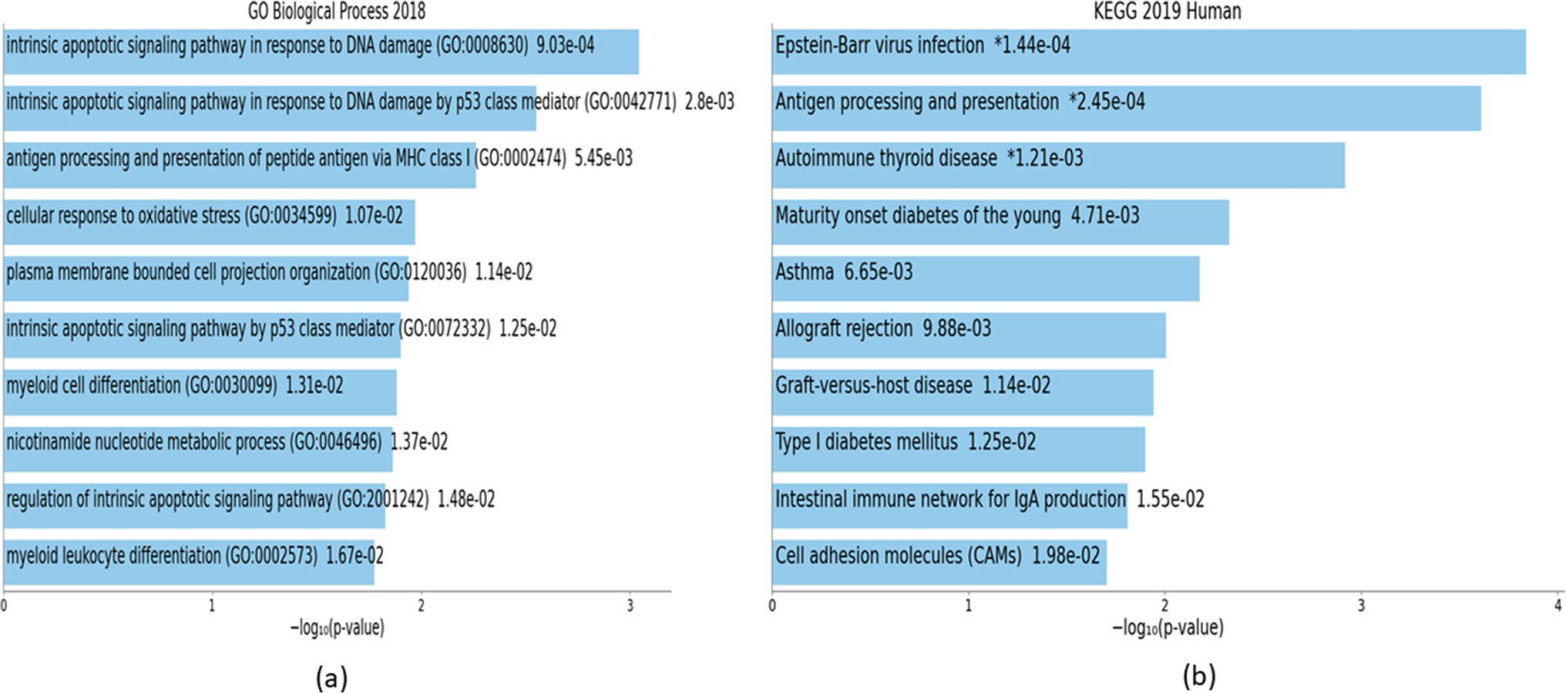
Downstream analyses of highly perturbed genes (*n* = 79) associated with type 2 diabetes in different tissues of human adults: **a** GO biological processes, **b** KEGG pathways

**Fig. 11:**
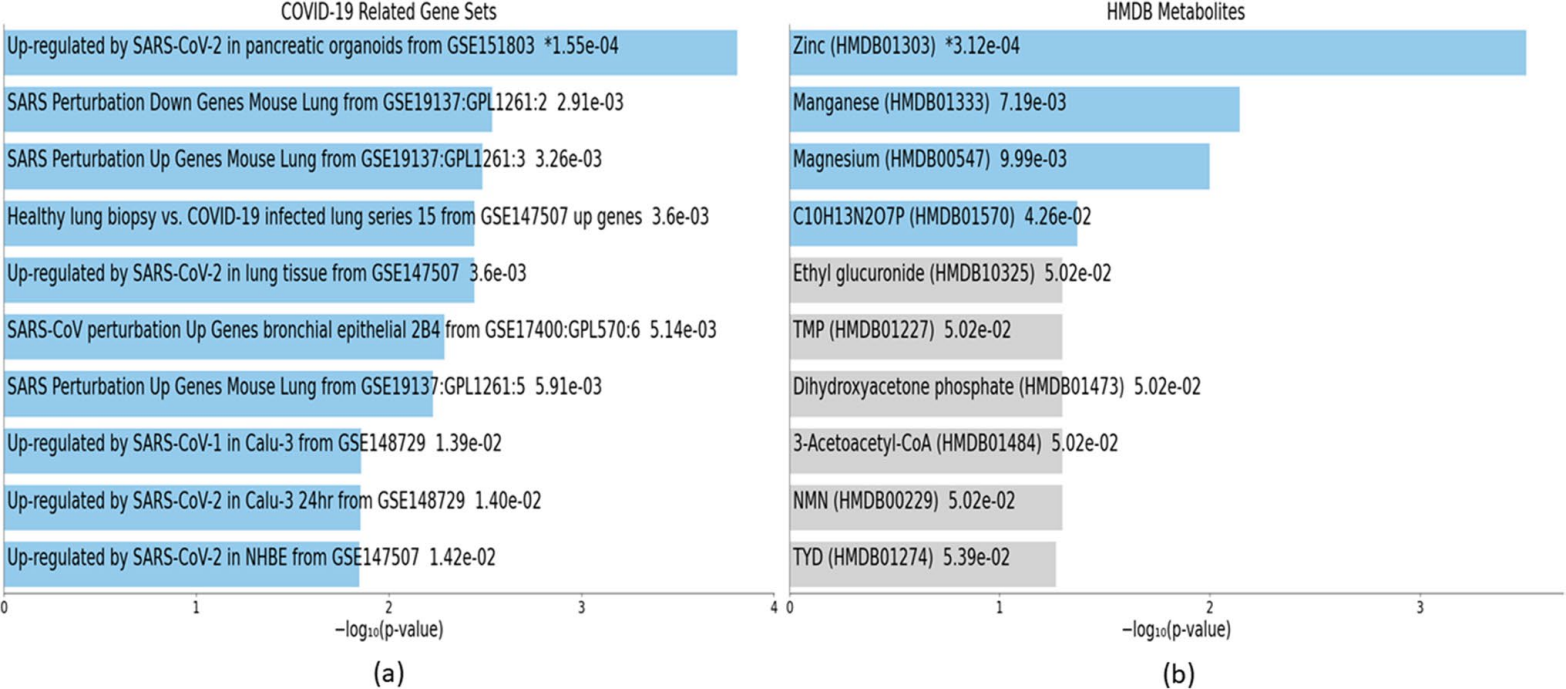
Downstream analyses of highly perturbed genes (*n* = 79) associated with type 2 diabetes in different tissues of human adults: **a** COVID-19 related gene sets, **b** HMDB metabolites

**Fig. 12:**
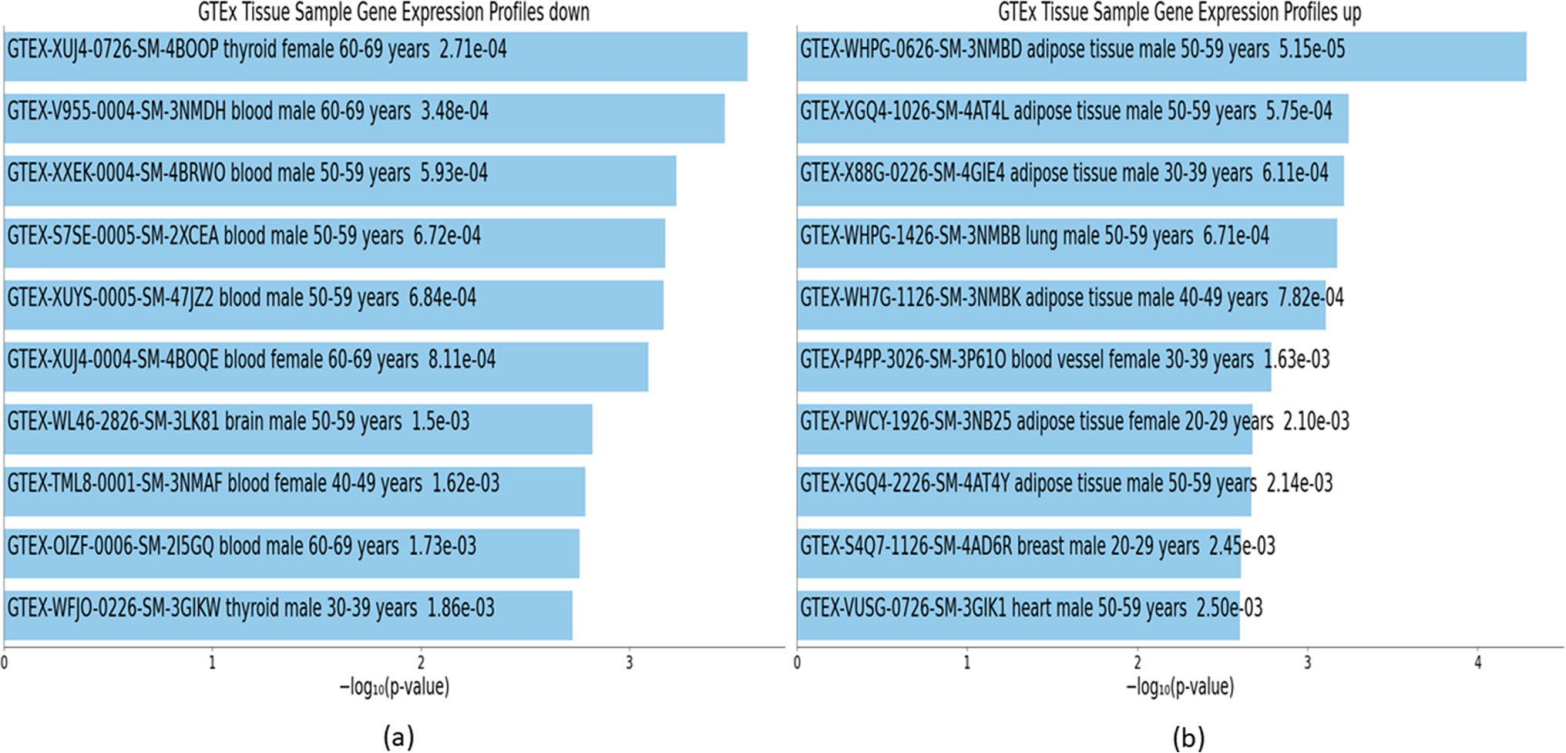
Downstream analyses of highly perturbed genes (*n* = 79) associated with type 2 diabetes in different tissues of human adults: **a** GTEx tissue sample gene expression profiles down, **b** GTEx tissue sample gene expression profiles up

**Fig. 13:**
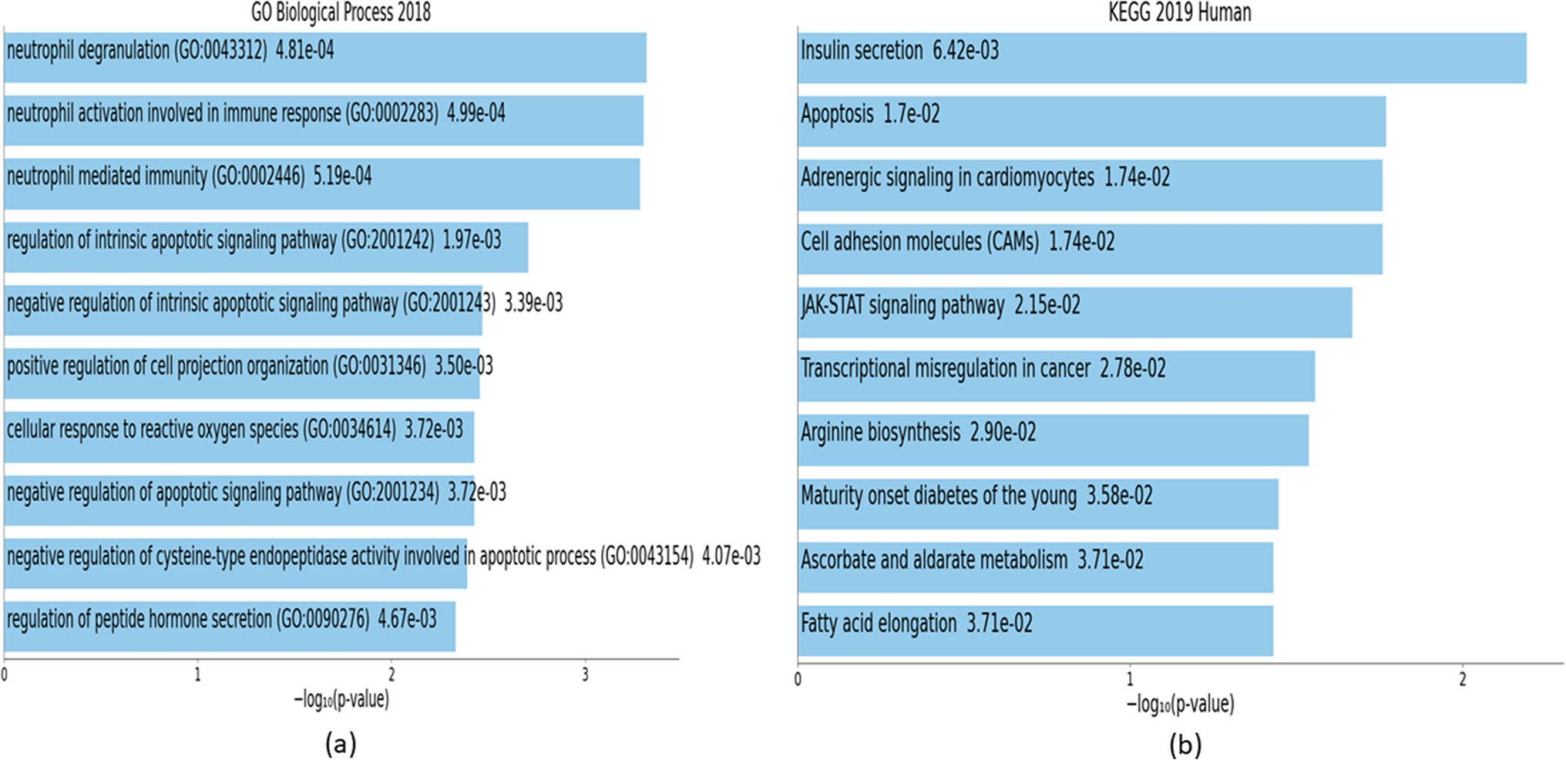
Downstream analyses of hub genes (*n* = 28) associated with type 2 diabetes in different tissues of human adults: **a** GO biological processes, **b** KEGG pathways

**Fig. 14:**
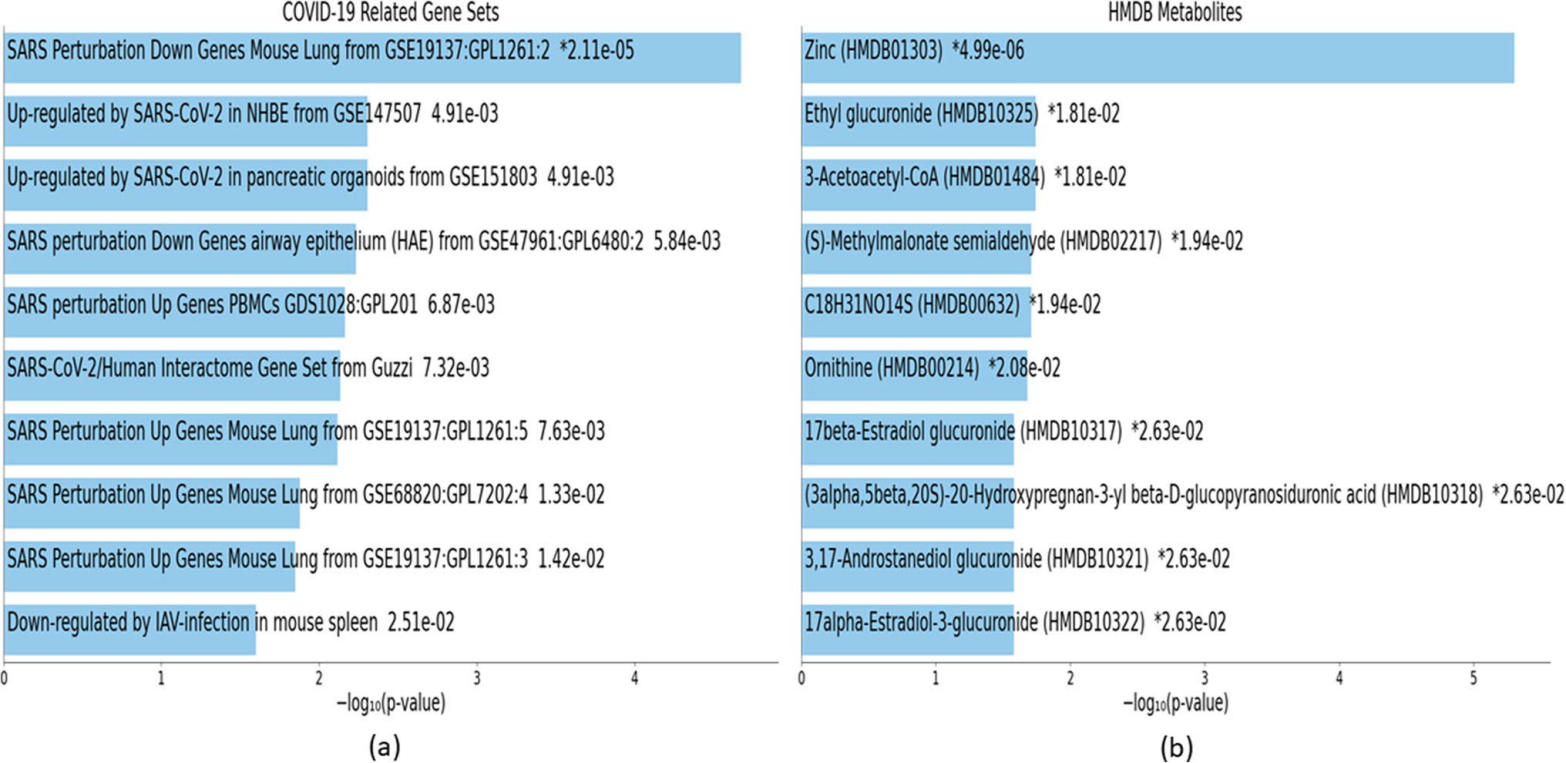
Downstream analyses of hub genes (*n* = 28) associated with type 2 diabetes in different tissues of human adults: **a** COVID-19-related gene sets, **b** HMDB metabolites

**Fig. 15:**
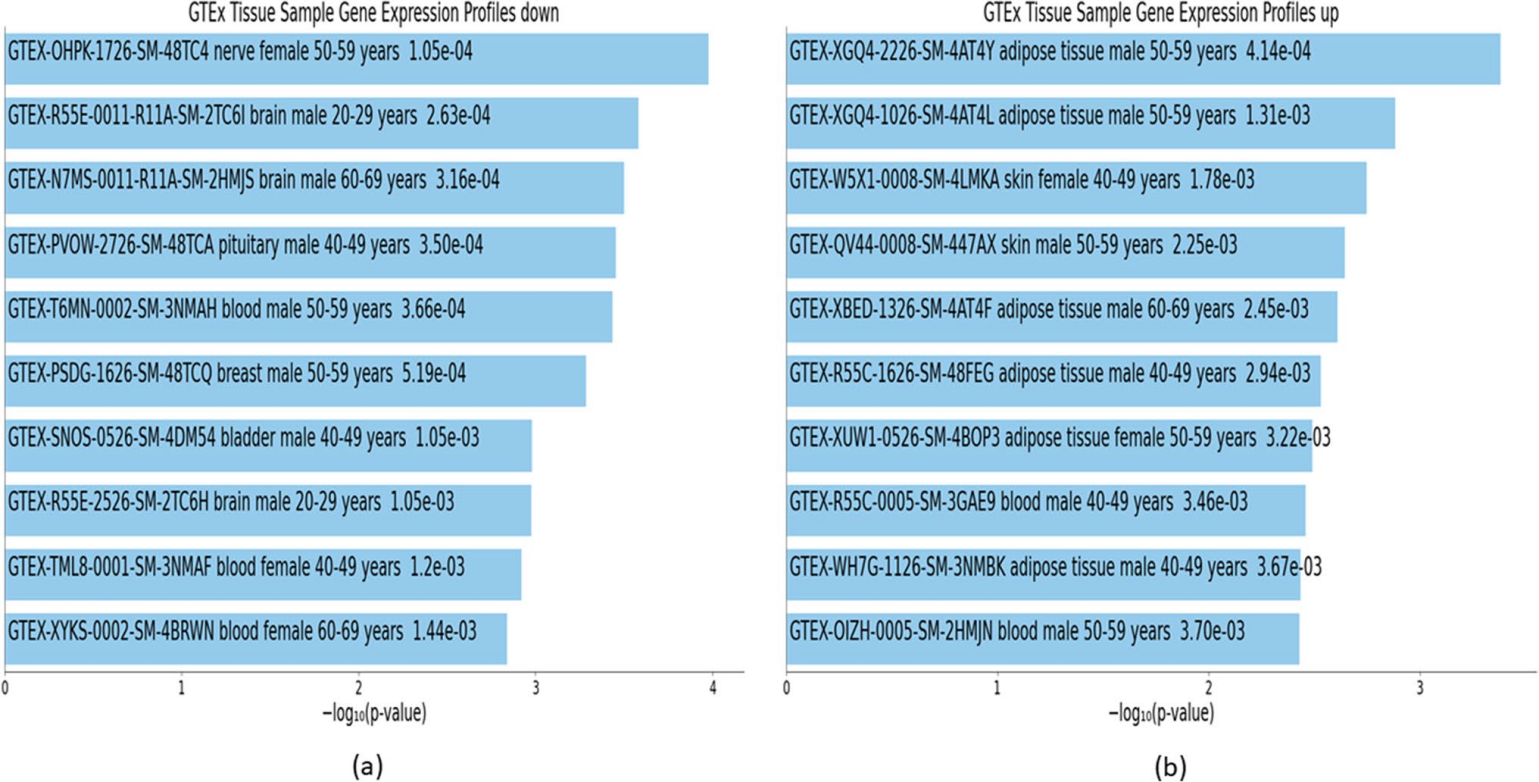
Downstream analyses of hub genes (*n* = 28) associated with type 2 diabetes in different tissues of human adults: **a** GTEx tissue sample gene expression profiles down, **b** GTEx tissue sample gene expression profiles up

Findings from downstream analyses of highly perturbed genes are summarized in Tables 4, 5, 6 and illustrated in Figs. 10, 11, 12 with details in Online Resource 10. Results from downstream analyses of hub genes are presented in Tables 7, 8, 9 and visualized in Figs. 13, 14, 15 with details in Online Resource 11.

**Table 4:** Downstream analyses of highly perturbed genes (*n* = 79) associated with type 2 diabetes in different tissues of human adults: GO biological processes and KEGG pathways

Term	Overlap	*p* value	Adjusted *p* value	Odds ratio	Combined score	Genes	Regulation in T2D
**Ontologies: GO biological processes**							
Intrinsic apoptotic signaling pathway in response to DNA damage (GO:0,008,630)	3/48	9.03E-04	0.230983	17.43509	122.2228	*DYRK2; BCL3; MCL1*	-
Intrinsic apoptotic signaling pathway in response to DNA damage by p53 class mediator (GO:0,042,771)	2/20	0.002795	0.230983	28.72006	168.8714	*DYRK2; BCL3*	-
Antigen processing and presentation of peptide antigen via MHC class I (GO:0,002,474)	2/28	0.005448	0.230983	19.87512	103.5986	*ERAP2; TAPBP*	Up
Cellular response to oxidative stress (GO:0,034,599)	3/115	0.010668	0.230983	6.981555	31.69995	*MMP9; DHRS2; SOD3*	-
Plasma membrane bounded cell projection organization (GO:0,120,036)	3/118	0.011436	0.230983	6.798398	30.39569	*CNTNAP2; STMN2; NPTX2*	-
Intrinsic apoptotic signaling pathway by p53 class mediator (GO:0,072,332)	2/43	0.012527	0.230983	12.59424	55.1606	*DYRK2; BCL3*	-
Myeloid cell differentiation (GO:0,030,099)	2/44	0.013091	0.230983	12.29375	53.30386	*DYRK3; ZBTB16*	Up
Nicotinamide nucleotide metabolic process (GO:0,046,496)	2/45	0.013665	0.230983	12.00725	51.54622	*NMNAT2; ALDOB*	-
Regulation of intrinsic apoptotic signaling pathway (GO:2,001,242)	2/47	0.014845	0.230983	11.47244	48.2997	*MMP9; MCL1*	Up
Myeloid leukocyte differentiation (GO:0,002,573)	2/50	0.016695	0.230983	10.75379	44.01114	*MMP9; DHRS2*	-
**Pathways: KEGG**							
Epstein–Barr virus infection	6/201	0.000144	0.012503	8.31444	73.51986	*IFNA7; HLA-DRB5; HLA-DRB4; ENTPD3; TAP2; TAPBP*	-
Antigen processing and presentation	4/77	0.000245	0.012503	14.50082	120.5547	*HLA-DRB5; HLA-DRB4; TAP2; TAPBP*	-
Autoimmune thyroid disease	3/53	0.001205	0.040983	15.68763	105.436	*IFNA7; HLA-DRB5; HLA-DRB4*	Down
Maturity-onset diabetes of the young (MODY)	2/26	0.004708	0.12006	21.53355	115.3862	*SLC2A2; IAPP*	Down
Asthma	2/31	0.006651	0.135681	17.81639	89.31323	*HLA-DRB5; HLA-DRB4*	Down
Allograft rejection	2/38	0.009878	0.159725	14.34704	66.24726	*HLA-DRB5; HLA-DRB4*	Down
Graft-versus-host disease	2/41	0.011434	0.159726	13.24142	59.20461	*HLA-DRB5; HLA-DRB4*	Down
Type I diabetes mellitus (T1DM)	2/43	0.012527	0.159726	12.59423	55.1606	*HLA-DRB5; HLA-DRB4*	Down
Intestinal immune network for IgA production	2/48	0.015452	0.175118	11.22247	46.79817	*HLA-DRB5; HLA-DRB4*	Down
Cell adhesion molecules (CAMs)	3/145	0.019771	0.181561	5.49824	21.5726	*CNTNAP2; HLA-DRB5; HLA-DRB4*	-

*GO*, gene ontology; *KEGG*, Kyoto Encyclopedia of Genes and Genomes; *T2D*, type 2 diabetes

**Table 5:** Downstream analyses of highly perturbed genes (*n* = 79) associated with type 2 diabetes in different tissues of human adults: COVID-19-related gene sets and HMDB metabolites

Term	Overlap	*p* value	Adjusted *p* value	Odds ratio	Combined score	Genes	Regulation in T2D
**Diseases: COVID-19-related gene sets**							
Upregulated by SARS-CoV-2 in pancreatic organoids from GSE151803	9/500	0.000155	0.01841	5.08787	44.64083	*TMEM37; PPP1R1A; VNN2; BCL3; TAP2; ELFN1; MMP9; DHRS2; SCN1B*	-
SARS perturbation down genes mouse lung from GSE19137:GPL1261:2	5/246	0.002908	0.08566	5.51755	32.22428	*PCOLCE2; RPL14; HADH; CTSC; MCL1*	-
SARS perturbation up genes mouse lung from GSE19137:GPL1261:3	6/366	0.003262	0.08566	4.46598	25.56907	*SLC9A3R2; HLA-DRB5; U2AF2; ZBTB16; RPL14; MCL1*	-
Healthy lung biopsy vs. COVID-19 infected lung series 15 from GSE147507 up genes	7/500	0.003599	0.08566	3.8313	21.55896	*POPDC3; HLA-DRB5; TNFAIP6; VNN2; BCL3; RNF19B; CTSC*	-
Upregulated by SARS-CoV-2 in lung tissue from GSE147507	7/500	0.003599	0.08566	3.8313	21.55896	*POPDC3; HLA-DRB5; TNFAIP6; VNN2; BCL3; RNF19B; CTSC*	-
SARS-CoV perturbation up genes bronchial epithelial 2B4 from GSE17400:GPL570:6	6/402	0.005143	0.100484	4.05251	21.35735	*ERAP2; VNN2; TAP2; RNF19B; PPM1K; DHRS2*	-
SARS perturbation up genes mouse lung from GSE19137:GPL1261:5	5/291	0.005911	0.100484	4.63877	23.80141	*HLA-DRB5; U2AF2; ZBTB16; RPL14; MCL1*	-
Upregulated by SARS-CoV-1 in Calu-3 from GSE148729	6/498	0.013921	0.16867	3.24574	13.87347	*APOL4; EGR2; TAP2; RNF19B; PPM1K; DHRS2*	-
Upregulated by SARS-CoV-2 in Calu-3 24 h from GSE148729	6/499	0.014047	0.16867	3.23899	13.81541	*APOL4; EGR2; ERAP2; TAP2; RNF19B; PPM1K*	-
Upregulated by SARS-CoV-2 in NHBE from GSE147507	6/500	0.014174	0.16867	3.23227	13.75766	*ENTPD3; BCL3; TAP2; PI3; MMP9; CTSC*	-
**Miscellaneous: HMDB metabolites**							
Zinc (HMDB01303)	4/82	0.000312	0.025909	13.56786	109.52001	*ZBTB16; ALDOB; MMP9; SOD3*	Up
Manganese (HMDB01333)	4/193	0.007193	0.127982	5.56811	27.4768	*ARG2; DYRK2; PPM1K; SOD3*	-
Magnesium (HMDB00547)	6/463	0.009991	0.127982	3.50061	16.12387	*DYRK3; DYRK2; ENTPD3; PPM1K; NMNAT2; MARK1*	-
C10H13N2O7P (HMDB01570)	1/11	0.042612	0.127982	25.52692	80.55315	*ENTPD3*	Down

**Table 6:** Downstream analyses of highly perturbed genes (*n* = 79) associated with type 2 diabetes in different tissues of human adults: GTEx tissue sample gene expression profiles

Term	Overlap	*p* value	Adjusted *p* value	Odds ratio	Combined score	Genes	Regulation in T2D
**Cell types: GTEx tissue sample gene expression profiles down**							
GTEX-XUJ4-0726-SM-4BOOP thyroid female 60–69 years	4/79	0.00027	0.3129	14.1127	115.93606	*HLA-DRB5; STMN2; RNF19B; DHRS2*	-
GTEX-V955-0004-SM-3NMDH blood male 60–69 years	23/2789	0.00034	0.3129	2.54729	20.28253	*CNTFR; POPDC3; RASL11B; PCOLCE2; ZBTB16; C14ORF132; NMNAT2; ASCL2; SOD3; POMZP3; SLC9A3R2; APOL4; ISLR; PPP1R1A; CRTAC1; PHACTR3; PI3; NPTX2; ZNF423; PVRL2; SCN1B; PRIMA1; MARK1*	-
GTEX-XXEK-0004-SM-4BRWO blood male 50–59 years	24/3085	0.00059	0.3129	2.40349	17.85933	*CNTFR; CNTNAP2; SNAP25; POPDC3; ARG2; RASL11B; ENTPD3; PCOLCE2; ZBTB16; XYLT1; C14ORF132; NMNAT2; ELFN1; ASCL2; SOD3; SLC9A3R2; ISLR; PPP1R1A; CRTAC1; NPTX2; ZNF423; PVRL2; SCN1B; MARK1*	-
GTEX-S7SE-0005-SM-2XCEA blood male 50–59 years	23/2919	0.00067	0.3129	2.41451	17.63845	*POPDC3; ARG2; RASL11B; ENTPD3; PCOLCE2; NMNAT2; LARP4; ELFN1; SOD3; SLC9A3R2; TMEM37; ISLR; ESPNL; KIAA1279; ENPP2; PHACTR3; HADH; NPTX2; ZNF423; PVRL2; SCN1B; PRIMA1; MARK1*	-
GTEX-XUYS-0005-SM-47JZ2 blood male 50–59 years	24/3115	0.00068	0.3129	2.37593	17.31639	*CNTFR; SNAP25; POPDC3; DYRK3; RASL11B; ENTPD3; PCOLCE2; ZBTB16; NMNAT2; LARP4; SOD3; SLC9A3R2; TMEM37; ISLR; KIAA1279; PPP1R1A; CRTAC1; ENPP2; HADH; NPTX2; ZNF423; PHLDA1; PVRL2; MARK1*	-
GTEX-XUJ4-0004-SM-4BOQE blood female 60–69 years	25/3348	0.00081	0.3129	2.31245	16.45932	*SNAP25; PCOLCE2; STMN2; ELFN1; ASCL2; SLC9A3R2; APOL4; ISLR; CRTAC1; PHACTR3; PI3; ZNF423; PHLDA1; SCN1B; PRIMA1; MARK1; ARG2; ENTPD3; ZBTB16; C14ORF132; NMNAT2; SOD3; PPP1R1A; ALDOB; PVRL2*	-
GTEX-WL46-2826-SM-3LK81 brain male 50–59 years	17/1966	0.00149	0.33149	2.52838	16.44486	*DYRK3; EGR2; MCOLN3; DYRK2; TNFAIP6; PCOLCE2; ERAP2; MMP9; ASCL2; SOD3; APOL4; ISLR; VNN2; BCL3; PVRL2; CTSC; MCL1*	-
GTEX-TML8-0001-SM-3NMAF blood female 40–49 years	22/2914	0.00161	0.33149	2.27268	14.60663	*CNTFR; RASL11B; ENTPD3; PCOLCE2; ZBTB16; STMN2; C14ORF132; NMNAT2; ASCL2; SOD3; SLC9A3R2; APOL4; ISLR; PPP1R1A; CRTAC1; ALDOB; ZNF423; PHLDA1; PVRL2; SCN1B; PRIMA1; MARK1*	-
GTEX-OIZF-0006-SM-2I5GQ blood male 60–69 years	32/5000	0.00172	0.33149	2.04927	13.03763	*CNTFR; POPDC3; DYRK3; MTRR; RASL11B; ELFN1; POMZP3; SLC9A3R2; APOL4; ISLR; CRTAC1; ENPP2; NAALAD2; HADH; NPTX2; ZNF423; PHLDA1; PAAF1; PRIMA1; MARK1; EGR2; MCOLN3; ENTPD3; ZBTB16; C14ORF132; NMNAT2; LARP4; SOD3; TMEM37; KIAA1279; PPP1R1A; PVRL2*	-
GTEX-WFJO-0226-SM-3GIKW thyroid male 30–39 years	5/222	0.00186	0.33149	6.13526	38.55908	*SNAP25; TNFAIP6; STMN2; ALDOB; PI3*	-
**Cell types: GTEx tissue sample gene expression profiles up**							
GTEX-WHPG-0626-SM-3NMBD adipose tissue male 50–59 years	12/778	0.00005	0.147791	4.47878	44.22047	*CNTFR; TMEM37; APOL4; DYRK3; EGR2; HLA-DRB5; PCOLCE2; BCL3; ENPP2; HADH; ZNF423; MCL1*	-
GTEX-XGQ4-1026-SM-4AT4L adipose tissue male 50–59 years	9/598	0.00057	0.448488	4.21994	31.48829	*SLC9A3R2; CNTFR; APOL4; EGR2; PPP1R1A; BCL3; HADH; ELFN1; MCL1*	-
GTEX-X88G-0226-SM-4GIE4 adipose tissue male 30–39 years	11/872	0.00061	0.448488	3.58099	26.50301	*TMEM37; APOL4; ISLR; EGR2; TNFAIP6; PCOLCE2; ERAP2; BCL3; TAP2; CTSC; MCL1*	-
GTEX-WHPG-1426-SM-3NMBB lung male 50–59 years	13/1178	0.00067	0.44848	3.17113	23.17132	*HLA-DRB5; MTRR; TAP2; RNF19B; MMP9; TAPBP; SLC9A3R2; APOL4; VNN2; CRTAC1; BCL3; CTSC; MCL1*	-
GTEX-WH7G-1126-SM-3NMBK adipose tissue male 40–49 years	10/758	0.00078	0.448488	3.71483	26.5752	*CNTFR; APOL4; DYRK3; TNFAIP6; PCOLCE2; PPP1R1A; BCL3; HADH; ELFN1; MCL1*	-
GTEX-P4PP-3026-SM-3P61O blood vessel female 30–39 years	7/434	0.00163	0.71722	4.43852	28.4846	*SLC9A3R2; CNTFR; APOL4; EGR2; RASL11B; PCOLCE2; ZNF423*	-
GTEX-PWCY-1926-SM-3NB25 adipose tissue female 20–29 years	10/865	0.0021	0.71722	3.23179	19.92596	*SLC9A3R2; CNTFR; TMEM37; APOL4; PCOLCE2; PPP1R1A; NAALAD2; HADH; ZNF423; LARP4*	-
GTEX-XGQ4-2226-SM-4AT4Y adipose tissue male 50–59 years	9/722	0.00213	0.717225	3.46367	21.29601	*SLC9A3R2; CNTFR; TMEM37; APOL4; PPP1R1A; ZBTB16; XYLT1; HADH; ZNF423*	-
GTEX-S4Q7-1126-SM-4AD6R breast male 20–29 years	9/737	0.00245	0.717225	3.38965	20.37124	*CNTFR; TMEM37; APOL4; PCOLCE2; PPP1R1A; ENPP2; HADH; ELFN1; POMZP3*	-
GTEX-VUSG-0726-SM-3GIK1 heart male 50–59 years	10/886	0.0025	0.7172	3.15085	18.87721	*POPDC3; APOL4; DYRK3; DYRK2; RASL11B; PCOLCE2; FUT11; COG2; HADH; ELFN1*	-

**Table 7:** Downstream analyses of hub genes (*n* = 28) associated with type 2 diabetes in different tissues of human adults: GO biological processes and KEGG pathways

Term	Overlap	*p* value	Adjusted *p* value	Odds ratio	Combined score	Genes	Regulation in T2D
**Ontologies: GO biological processes**							
Neutrophil degranulation (GO:0,043,312)	5/479	0.000481	0.061879	8.94239	68.31934	*SNAP25; TNFAIP6; ARG1; MMP9; CTSC*	-
Neutrophil activation involved in immune response (GO:0,002,283)	5/483	0.000499	0.061878	8.86574	67.39777	*SNAP25; TNFAIP6; ARG1; MMP9; CTSC*	-
Neutrophil-mediated immunity (GO:0,002,446)	5/487	0.000518	0.061878	8.79036	66.49468	*SNAP25; TNFAIP6; ARG1; MMP9; CTSC*	-
Regulation of intrinsic apoptotic signaling pathway (GO:2,001,242)	2/47	0.001965	0.081572	34.06324	212.28967	*MMP9; MCL1*	Up
Negative regulation of intrinsic apoptotic signaling pathway (GO:2,001,243)	2/62	0.003393	0.081572	25.5282	145.15226	*MMP9; MCL1*	Up
Positive regulation of cell projection organization (GO:0,031,346)	2/63	0.003501	0.081572	25.10844	141.97721	*STMN2; SCN1B*	-
Cellular response to reactive oxygen species (GO:0,034,614)	2/65	0.003722	0.081572	24.30891	135.96666	*DPEP1; MMP9*	-
Negative regulation of apoptotic signaling pathway (GO:2,001,234)	2/65	0.003722	0.081572	24.30891	135.96666	*MMP9; MCL1*	Up
Negative regulation of cysteine-type endopeptidase activity involved in apoptotic process (GO:0,043,154)	2/68	0.004066	0.081572	23.20046	127.71724	*DPEP1; MMP9*	-
Regulation of peptide hormone secretion (GO:0,090,276)	2/73	0.004671	0.081572	21.56121	115.70462	*SNAP25; SLC2A2*	Down
**Pathways: KEGG**							
Insulin secretion	2/86	0.006424	0.166054	18.21245	91.9304	*SNAP25; SLC2A2*	Down
Apoptosis	2/143	0.016992	0.166054	10.81887	44.08705	*CTSC; MCL1*	-
Adrenergic signaling in cardiomyocytes	2/145	0.017442	0.166054	10.66648	43.18703	*PPP1R1A; SCN1B*	-
Cell adhesion molecules (CAMs)	2/145	0.017442	0.166054	10.66648	43.18703	*CNTNAP2; CD226*	-
JAK-STAT signaling pathway	2/162	0.021472	0.166054	9.525	36.58529	*CNTFR; MCL1*	Up
Transcriptional mis-regulation in cancer	2/186	0.027752	0.166054	8.27257	29.65238	*ZBTB16; MMP9*	Up
Arginine biosynthesis	1/21	0.029006	0.166054	36.94814	130.80559	*ARG1*	-
Maturity-onset diabetes of the young (MODY)	1/26	0.035792	0.166054	29.55111	98.40622	*SLC2A2*	Down
Ascorbate and aldarate metabolism	1/27	0.037143	0.166054	28.4131	93.5634	*UGT2B7*	Down
Fatty acid elongation	1/27	0.037143	0.166054	28.4131	93.5634	*HADH*	Down

*GO*, gene ontology; *KEGG*, Kyoto Encyclopedia of Genes and Genomes; *T2D*, = type 2 diabetes

**Table 8:** Downstream analyses of hub genes (*n* = 28) associated with type 2 diabetes in different tissues of human adults: COVID-19-related gene sets and HMDB metabolites

Term	Overlap	*p* value	Adjusted *p* value	Odds ratio	Combined score	Genes	Regulation in T2D
**Diseases: COVID-19-related gene sets**							
SARS perturbation down genes mouse lung from GSE19137:GPL1261:2	5/246	0.000021	0.002066	17.79812	191.63596	*RPL14; DPEP1; HADH; CTSC; MCL1*	-
Upregulated by SARS-CoV-2 in NHBE from GSE147507	4/500	0.004909	0.106835	6.54435	34.79441	*PPP1R15A; TAP1; MMP9; CTSC*	-
Upregulated by SARS-CoV-2 in pancreatic organoids from GSE151803	4/500	0.004909	0.106835	6.54435	34.79441	*PPP1R1A; TAP1; MMP9; SCN1B*	-
SARS perturbation down genes airway epithelium (HAE) from GSE47961:GPL6480:2	3/264	0.005837	0.106835	9.06253	46.61365	*RASL11B; ENPP2; MMP9*	-
SARS perturbation up genes PBMCs GDS1028:GPL201	3/280	0.006865	0.106835	8.53213	42.50101	*PPP1R15A; ARG1; MMP9*	-
SARS-CoV-2/human interactome gene set from Guzzi	2/92	0.007319	0.106835	16.99316	83.55855	*ISLR; MCL1*	Up
SARS perturbation up genes mouse lung from GSE19137:GPL1261:5	3/291	0.007631	0.106835	8.20166	39.98742	*ZBTB16; RPL14; MCL1*	-
SARS perturbation up genes mouse lung from GSE68820:GPL7202:4	3/357	0.013276	0.142647	6.65016	28.74044	*TNFAIP6; ARG1; TAP1*	-
SARS perturbation up genes mouse lung from GSE19137:GPL1261:3	3/366	0.014189	0.142647	6.48231	27.58368	*ZBTB16; RPL14; MCL1*	-
Downregulated by IAV-infection in mouse spleen	3/455	0.025142	0.142647	5.1823	19.08734	*ISLR; ENPP2; SOD3*	-
**Miscellaneous: HMDB metabolites**							
Zinc (HMDB01303)	4/82	4.99E-06	0.000264	42.50854	518.98916	*ZBTB16; DPEP1; SOD3; MMP9*	-
Ethyl glucuronide (HMDB10325)	1/13	0.018053	0.035712	61.60493	247.30909	*UGT2B7*	Down
3-Acetoacetyl-CoA (HMDB01484)	1/13	0.018053	0.035712	61.60493	247.30909	*HADH*	Down
(S)-Methylmalonate semialdehyde (HMDB02217)	1/14	0.019428	0.035712	56.86324	224.09815	*HADH*	Down
C18H31NO14S (HMDB00632)	1/14	0.019428	0.035712	56.86324	224.09815	*XYLT1*	Up
Ornithine (HMDB00214)	1/15	0.020802	0.035712	52.79894	204.47351	*ARG1*	-
17beta-Estradiol glucuronide (HMDB10317)	1/19	0.026278	0.035712	41.05761	149.40806	*UGT2B7*	Down
(3alpha,5beta,20S)-20-Hydroxypregnan-3-yl beta-D-glucopyranosiduronic acid (HMDB10318)	1/19	0.026278	0.035712	41.05761	149.40806	*UGT2B7*	Down
3,17-Androstanediol glucuronide (HMDB10321)	1/19	0.026278	0.035712	41.05761	149.40806	*UGT2B7*	Down
17alpha-Estradiol-3-glucuronide (HMDB10322)	1/19	0.026278	0.035712	41.05761	149.40806	*UGT2B7*	Down

**Table 9:** Downstream analyses of hub genes (*n* = 28) associated with type 2 diabetes in different tissues of human adults: GTEx tissue sample gene expression profiles

Term	Overlap	*p* value	Adjusted *p* value	Odds ratio	Combined score	Genes	Regulation in T2D
**Cell types: GTEx tissue sample gene expression profiles down**							
GTEX-OHPK-1726-SM-48TC4 nerve female 50–59 years	4/178	0.00010	0.140263	18.9636	173.71976	*SNAP25; CNTNAP2; ARG1; CD226*	-
GTEX-R55E-0011-R11A-SM-2TC6I brain male 20–29 years	8/1275	0.00026	0.140263	5.905288	48.68668	*TNFAIP6; ZBTB16; RPL14; TAP1; DPEP1; MMP9; CTSC; MCL1*	-
GTEX-N7MS-0011-R11A-SM-2HMJS brain male 60–69 years	10/2072	0.00031	0.140263	4.82541	38.89372	*ISLR; TNFAIP6; ZBTB16; XYLT1; TAP1; CD226; SOD3; MMP9; CTSC; MCL1*	-
GTEX-PVOW-2726-SM-48TCA pituitary male 40–49 years	5/447	0.00035	0.140263	9.60554	76.42437	*TNFAIP6; ARG1; ZBTB16; XYLT1; MCL1*	-
GTEX-T6MN-0002-SM-3NMAH blood male 50–59 years	12/2999	0.00036	0.140263	4.26473	33.74814	*CNTNAP2; CNTFR; ISLR; RASL11B; PPP1R1A; ZBTB16; STMN2; DPEP1; ZNF423; PHLDA1; SOD3; SCN1B*	-
GTEX-PSDG-1626-SM-48TCQ breast male 50–59 years	5/487	0.00051	0.165673	8.79036	66.49468	*SNAP25; CNTNAP2; RASL11B; STMN2; CD226*	-
GTEX-SNOS-0526-SM-4DM54 bladder male 40–49 years	3/144	0.00104	0.232779	16.87744	115.77124	*TNFAIP6; PPP1R1A; STMN2*	-
GTEX-R55E-2526-SM-2TC6H brain male 20–29 years	5/570	0.00105	0.232779	7.4671	51.1853	*TNFAIP6; ZBTB16; TAP1; DPEP1; CTSC*	-
GTEX-TML8-0001-SM-3NMAF blood female 40–49 years	11/2914	0.00119	0.232779	3.80456	25.59278	*CNTFR; ISLR; RASL11B; PPP1R1A; ARG1; ZBTB16; STMN2; ZNF423; PHLDA1; SOD3; SCN1B*	-
GTEX-XYKS-0002-SM-4BRWN blood female 60–69 years	11/2979	0.001443	0.232779	3.70707	24.24791	*SNAP25; CNTNAP2; CNTFR; ISLR; RASL11B; PPP1R1A; ZBTB16; STMN2; ZNF423; SOD3; SCN1B*	-
**Cell types: GTEx tissue sample gene expression profiles up**							
GTEX-XGQ4-2226-SM-4AT4Y adipose tissue male 50–59 years	6/722	0.000413	0.606044	7.33469	57.14262	*CNTFR; PPP1R1A; ZBTB16; XYLT1; HADH; ZNF423*	-
GTEX-XGQ4-1026-SM-4AT4L adipose tissue male 50–59 years	5/598	0.001305	0.606044	7.10426	47.18102	*PPP1R15A; CNTFR; PPP1R1A; HADH; MCL1*	-
GTEX-W5X1-0008-SM-4LMKA skin female 40–49 years	6/955	0.001779	0.606044	5.466902	34.61318	*ISLR; RPL14; ENPP2; XYLT1; DPEP1; CTSC*	-
GTEX-QV44-0008-SM-447AX skin male 50–59 years	8/1765	0.002254	0.606044	4.14684	25.2735	*PPP1R15A; ISLR; RASL11B; ENPP2; XYLT1; PHLDA1; CTSC; MCL1*	-
GTEX-XBED-1326-SM-4AT4F adipose tissue male 60–69 years	5/690	0.002448	0.606044	6.120913	36.80156	*PPP1R15A; CNTFR; ENPP2; MMP9; MCL1*	-
GTEX-R55C-1626-SM-48FEG adipose tissue male 40–49 years	5/720	0.002943	0.606044	5.854971	34.12304	*CNTFR; PPP1R1A; ZBTB16; ENPP2; XYLT1*	-
GTEX-XUW1-0526-SM-4BOP3 adipose tissue female 50–59 years	5/735	0.003217	0.606044	5.730196	32.88661	*CNTFR; PPP1R1A; ZBTB16; ENPP2; HADH*	-
GTEX-R55C-0005-SM-3GAE9 blood male 40–49 years	6/1090	0.003461	0.606044	4.752096	26.92674	*TNFAIP6; ARG1; CD226; MMP9; CTSC; MCL1*	-
GTEX-WH7G-1126-SM-3NMBK adipose tissue male 40–49 years	5/758	0.003672	0.606044	5.54853	31.11036	*CNTFR; TNFAIP6; PPP1R1A; HADH; MCL1*	-
GTEX-OIZH-0005-SM-2HMJN blood male 50–59 years	6/1105	0.003703	0.606044	4.68351	26.221	*TNFAIP6; ARG1; TAP1; CD226; MMP9; MCL1*	-

### Enriched biological processes associated with highly perturbed genes and hub genes of T2D

Ontology analysis of highly perturbed genes identified 89 enriched biological processes (Online Resource 10). The top 10 enriched biological processes were intrinsic apoptotic signaling pathway in response to DNA damage (GO:0,008,630); intrinsic apoptotic signaling pathway in response to DNA damage by p53 class mediator (GO:0,042,771); antigen processing and presentation of peptide antigen via MHC class I (GO:0,002,474); cellular response to oxidative stress (GO:0,034,599); plasma membrane bounded cell projection organization (GO:0,120,036); intrinsic apoptotic signaling pathway by p53 class mediator (GO:0,072,332); myeloid cell differentiation (GO:0,030,099); nicotinamide nucleotide metabolic process (GO:0,046,496); regulation of intrinsic apoptotic signaling pathway (GO:2,001,242); and myeloid leukocyte differentiation (GO:0,002,573). Three of these, antigen processing and presentation of peptide antigen via MHC class I (GO:0,002,474); myeloid cell differentiation (GO:0,030,099), and regulation of intrinsic apoptotic signaling pathway (GO:2,001,242), were associated with upregulated genes in T2D (Table 4).

Ontological analysis of hub genes identified 179 enriched biological processes (Online Resource 11). Top 10 enriched biological processes were neutrophil degranulation (GO:0,043,312); neutrophil activation involved in immune response (GO:0,002,283); neutrophil-mediated immunity (GO:0,002,446); regulation of intrinsic apoptotic signaling pathway (GO:2,001,242); negative regulation of intrinsic apoptotic signaling pathway (GO:2,001,243); positive regulation of cell projection organization (GO:0,031,346); cellular response to reactive oxygen species (GO:0,034,614); negative regulation of apoptotic signaling pathway (GO:2,001,234); negative regulation of cysteine-type endopeptidase activity involved in apoptotic process (GO:0,043,154); and regulation of peptide hormone secretion (GO:0,090,276). Of these, three (regulation of intrinsic apoptotic signaling pathway (GO:2,001,242); negative regulation of intrinsic apoptotic signaling pathway (GO:2,001,243); negative regulation of apoptotic signaling pathway (GO:2,001,234)) were associated with upregulated genes in T2D, whereas one (regulation of peptide hormone secretion (GO:0,090,276)) was associated with downregulated genes in T2D (Table 6).

### Enriched KEGG pathways associated with highly perturbed genes and hub genes of T2D

As per KEGG analysis of highly perturbed genes, 21 pathways were enriched (Online Resource 10). The top 10 pathways were Epstein–Barr virus infection; antigen processing and presentation; autoimmune thyroid disease; maturity-onset diabetes of the young; asthma; allograft rejection; graft-versus-host disease; type 1 diabetes mellitus; intestinal immune network for IgA production; and cell adhesion molecules (CAMs). Seven of these (autoimmune thyroid disease; maturity-onset diabetes of the young; asthma; allograft rejection; graft-versus-host disease; type 1 diabetes mellitus; intestinal immune network for IgA production) were associated with downregulated genes in T2D (Table 4).

According to KEGG analysis of hub genes, 12 pathways were enriched (Online Resource 11). The top 10 pathways were insulin secretion; apoptosis; adrenergic signaling in cardiomyocytes; cell adhesion molecules (CAMs); JAK-STAT signaling pathway; transcriptional mis-regulation in cancer; arginine biosynthesis; maturity-onset diabetes of the young; ascorbate and aldarate metabolism; and fatty acid elongation. Four of these (insulin secretion; maturity-onset diabetes of the young; ascorbate and aldarate metabolism; fatty acid elongation) were associated with downregulated genes in T2D, while two pathways (JAK-STAT signaling pathway and transcriptional mis-regulation in cancer) were associated with upregulated genes in T2D (Table 6).

### COVID-19 related gene sets associated with highly perturbed genes and hub genes of T2D

Downstream analyses revealed 20 COVID-19-related gene sets associated with the highly perturbed genes of T2D (Online Resource 10), and the top 10 of these are visualized in Fig. 11a. There were 23 COVID-19-related gene sets associated with the hub genes of T2D (Online Resource 11), the top 10 of which are visualized in Fig. 14a.

### HMDB metabolites associated with highly perturbed genes and hub genes of T2D

Four HMDB metabolites (zinc (HMDB01303), manganese (HMDB01333), magnesium (HMDB00547), C10H13N2O7P (HMDB01570)) were associated with the highly perturbed genes of T2D (Online Resource 10). Of these, Zinc (HMDB01303) was associated with upregulated genes in T2D, while C10H13N2O7P (HMDB01570) was associated with downregulated genes in T2D (Table 5).

There were 45 HMDB metabolites associated with the hub genes of T2D (Online Resource 11). The top 10 metabolites were zinc (HMDB01303); ethyl glucuronide (HMDB10325); 3-acetoacetyl-CoA (HMDB01484); (S)-methylmalonate semialdehyde (HMDB02217); C18H31NO14S (HMDB00632); ornithine (HMDB00214); 17beta-estradiol glucuronide (HMDB10317); (3alpha,5beta,20S)-20-hydroxypregnan-3-yl beta-D-glucopyranosiduronic acid (HMDB10318); 3,17-androstanediol glucuronide (HMDB10321); and 17alpha-estradiol-3-glucuronide (HMDB10322). Seven of these (Ethyl glucuronide (HMDB10325); 3-acetoacetyl-CoA (HMDB01484); (S)-methylmalonate semialdehyde (HMDB02217); 17beta-estradiol glucuronide (HMDB10317); (3alpha,5beta,20S)-20-hydroxypregnan-3-yl beta-D-glucopyranosiduronic acid (HMDB10318); 3,17-androstanediol glucuronide (HMDB10321); and 17alpha-estradiol-3-glucuronide (HMDB10322)) were associated with downregulated genes in T2D. One metabolite (C18H31NO14S (HMDB00632)) was associated with upregulated genes of T2D (Table 8).

### Gene expression in different cell types associated with highly perturbed genes and hub genes of T2D

There were 168 downregulated GTEx profiles (Online Resource 10) associated with highly perturbed genes of T2D, the top 10 of which consisted of one brain, two thyroids, and seven blood tissue samples (Table 6 and Fig. 12). There were 77 upregulated GTEx profiles (Online Resource 10) associated with highly perturbed genes of T2D, the top 10 of which consisted of six adipose and one each of lung, breast, heart, and blood vessel tissue samples (Table 6 and Fig. 12).

There were 223 downregulated GTEx profiles (Online Resource 11) associated with hub genes of T2D, the top 10 of which consisted of five nervous system (three brain, one nerve, one pituitary), three blood, one breast, and one bladder tissue samples (Table 9 and Fig. 15). There were 178 upregulated GTEx profiles (Online Resource 11) associated with hub genes of T2D, the top 10 of which consisted of six adipose, two blood, and two skin tissue samples (Table 9 and Fig. 15).

## Discussion

In this study, we identified highly perturbed genes and hub genes associated with T2D in different tissues of adult humans, via an extensive bioinformatics analytic workflow. Downstream analyses revealed valuable insights on T2D pathogenesis, including associations with other diabetes phenotypes and COVID-19 and patterns of tissue-specific and tissue non-specific differential gene expression as well as pathophysiological manifestations such as those related to insulin action, immunity, and apoptosis. Salient findings of the study which contribute towards the understanding of the genetic basis of T2D are further discussed below. The comprehensive evidence synthesis approach with open-source gene expression data exemplified in this study could be replicated to gain high-level evidence synthesis for other clinical conditions.

### Patterns of differential gene expression in T2D

Our findings indicate that T2D seems rather a disorder of gene downregulation than upregulation, when the whole genome is considered. This is consistent with previous studies where a preponderance of gene downregulation was associated with T2D (Takematsu et al. 2020; Palsgaard et al. 2009). Also, hyperglycemia-induced global downregulation of gene expression in adipose and skeletal muscle tissues have been documented previously (Meugnier et al. 2007). A similar pattern has been observed in T1D (Yip et al. 2020) and other endocrine disorders such as polycystic ovary syndrome (Idicula-Thomas et al. 2020). In contrast, highly perturbed genes and hub genes associated with T2D, which might together constitute the candidate gene set critical for pathogenicity of T2D, were found to contain both up- and downregulated genes. This presentation suggests a more complex dysregulation at the crux of the GRN of T2D, involving actions and interactions between both repressed and augmented genes.

### Tissue-specific and tissue non-specific DEGs associated with T2D

Results of the present study indicate the predominance of tissue-specific DEGs in T2D. This supports the use of target tissue gene expression analysis as a viable avenue for identifying tissue-specific T2D biomarkers. A previous analysis integrating multiple tissue transcriptomics and PPI data to explore molecular biomarkers of T2D confirmed the presence of common signatures (Calimlioglu et al. 2015). We also observed common DEGs across different tissue types which can act as confluent molecular signatures of T2D. Identification of tissue-specific and non-specific molecular gene signatures of T2D facilitates downstream exploration of key pathways amenable to therapeutic targeting and drug repurposing efforts.

### Shared gene enrichment across diabetes phenotypes

As revealed by KEGG pathway analyses, both MODY and T1D were enriched pathways associated with highly perturbed genes of T2D, while MODY was also an enriched pathway associated with hub genes of T2D. Specifically, *SLC2A2* (*GLUT2*), and *IAPP* genes were commonly enriched in MODY, while *HLA-DRB4* and *HLA-DRB5* genes were underlying the enrichment with T1D. A gene expression meta-analysis also revealed the existence of possible pleiotropic mechanisms manifest via common gene signatures (*PGRMC1* and *HADH*) across different diabetes phenotypes (Mei et al. 2017).

Downregulation of *SLC2A2* is associated with not only T2D (Solimena et al. 2018) but also neonatal diabetes (Sansbury et al. 2012) and early childhood diabetes (Alhaidan et al. 2020), suggesting a likely role in insulin secretion. Amylin (*IAPP*), a gluco-modulatory hormone co-expressed with insulin by pancreatic β cells, is downregulated in both T1D and advanced T2D (Abedini et al. 2013), while amylin agonists are considered novel therapeutic agents for treating diabetes (Sonne et al. 2021). Moreover, human amylin plays a protective role against autoimmune diabetes inducing CD4 + Foxp3 + regulatory T cells (Zhang et al. 2018). Downregulation of *HLA-DRB4* in peripheral blood mononuclear cells is associated with T2D as well as dyslipidemia and periodontitis (Corbi et al. 2020), while a meta-analysis revealed that the lack of *HLA-DRB5* increased T2D risk (Jacobi et al. 2020). Of note, both *HLA-DRB4* and *HLA-DRB5* are associated with β cell autoantibodies and T1D (Zhao et al. 2016), with previous studies reporting that both T1D and T2D share HLA class II locus components (Jacobi et al. 2020). Interestingly, two of the hub genes of T2D found in our study (*MMP9, ARG1*) have been found as hub genes of T1D in a previous analysis (Yang et al. 2020). Collectively, these findings support some degree of shared genetic architecture between T2D and other diabetes pathologies.

### T2D as a disorder of insulin secretion and action

Downstream analyses provided insights into the characterization of T2D as a disorder of insulin secretion and action. We found that insulin secretion was the most significant KEGG pathway associated with hub genes, whereby two downregulated hub genes (*SLC2A2*, *SNAP25*) in T2D were underlying this enrichment. Zinc, the most significant HMDB metabolite associated with both highly perturbed genes and hub genes of T2D, is an essential element with key regulatory roles in insulin synthesis, storage, and secretion (Kim and Lee 2012). Other metabolomic markers associated with highly perturbed genes included magnesium which is necessary for insulin signaling (Piuri et al. 2021) as well as manganese which is involved in insulin synthesis and secretion (Chen et al. 2018). Together, these findings underscore the effects on insulin production and action as pivotal to T2D pathogenesis.

### Pathophysiological manifestations of T2D

#### *Apoptosis*

Downstream analyses revealed that multiple GO and KEGG pathways associated with apoptosis, including intrinsic apoptotic signaling pathway, were enriched in T2D. It is known that hyperglycemia-induced β cell apoptosis, a hallmark in T2D progression, occurs via intrinsic pathways causing reduced islet mass and metabolic abnormalities (Wali et al. 2013). Hyperglycemia-induced apoptosis has been reported to occur in other sites such as renal cells (Jung et al. 2012) and coronary arteries (Kageyama et al. 2011), indicating a possible role in disease progression and the onset of complications.

#### *Immunity*

Downstream analyses also revealed that multiple immunity-related GO and KEGG pathways, encompassing both innate and humoral immune responses, were enriched in T2D. These included multiple ontologies involving neutrophils and antigen processing and presentation as well as severe immune reactions such as graft-versus-host disease and allograft rejection. Impaired immunity in T2D and consequent susceptibility to infections and complications is frequently observed (Berbudi et al. 2020). A deeper understanding of the genomics underlying impaired immunity in T2D might provide opportunities to personalize the management of comorbidities and pharmacotherapy.

### COVID-19 and T2D

Epidemiological studies strongly suggest poorer prognosis of COVID-19 among people with T2D (Selvin and Juraschek, 2020), although underlying mechanisms are not well-understood (Apicella et al. 2020). Downstream analysis of highly perturbed genes and hub genes of T2D in the present study revealed a large number of enriched COVID-19-related gene sets, providing support for this putative link at a more granular level.

### Over- and under-expression of genes in different tissues associated with T2D

Downstream analysis of GTEx profiles identified tissues that are likely to demonstrate under- and over-expression of DEGs associated with T2D. Findings indicate that adipose tissue tends to over-express marker genes of T2D, while these might be under-expressed in other tissues such as those of the nervous system. These findings have implications for biomarker discovery and can guide further research on tissues which should be explored for identifying DEGs.

## Conclusions

Findings of this study contribute towards the understanding of the genetic basis of T2D, and further research is warranted to substantiate the molecular mechanisms underlying these findings which is fundamental to establishing precision T2D medicine initiatives. The proposed bioinformatics pipeline may have broader use as a judicious strategy to identify gene perturbations and pathophysiological mechanisms of other clinical conditions beyond T2D which ought to be validated in future research. Finally, this study describes an exemplary approach to applying comprehensive evidence synthesis using existing open-source gene expression data. Other researchers are encouraged to apply this methodology to obtain high-level evidence from existing multiple datasets, thereby getting the most value from existing bioinformatics sources.

## Supplementary Information

Below is the link to the electronic supplementary material.

Supplementary file1 (PDF 91 KB)

Supplementary file2 (XLSX 19 KB)

Supplementary file3 (XLSX 16 KB)

Supplementary file4 (XLSX 744 KB)

Supplementary file5 (XLSX 68 KB)

Supplementary file6 (XLSX 962 KB)

Supplementary file7 (XLSX 19 KB)

Supplementary file8 (XLSX 65 KB)

Supplementary file9 (XLSX 44 KB)

Supplementary file10 (XLSX 29 KB)

Supplementary file11 (XLSX 36.6 KB)

## Data Availability

Data used in this study are freely available at the National Center for Biotechnology Information Gene Expression Omnibus (NCBI GEO) portal: https://www.ncbi.nlm.nih.gov/geo/

## Code availability

All analytic codes are available on reasonable request from the corresponding author.
