# Supplementary figures and images for: Highly perturbed genes and hub genes associated with type 2 diabetes in different tissues of adult humans: a bioinformatics analytic workflow
Source: Funct Integr Genomics. 2022 Jul 5;22(5):1003–29. doi: 10.1007/s10142-022-00881-5 (PMC9255467; doi:10.1007/s10142-022-00881-5)

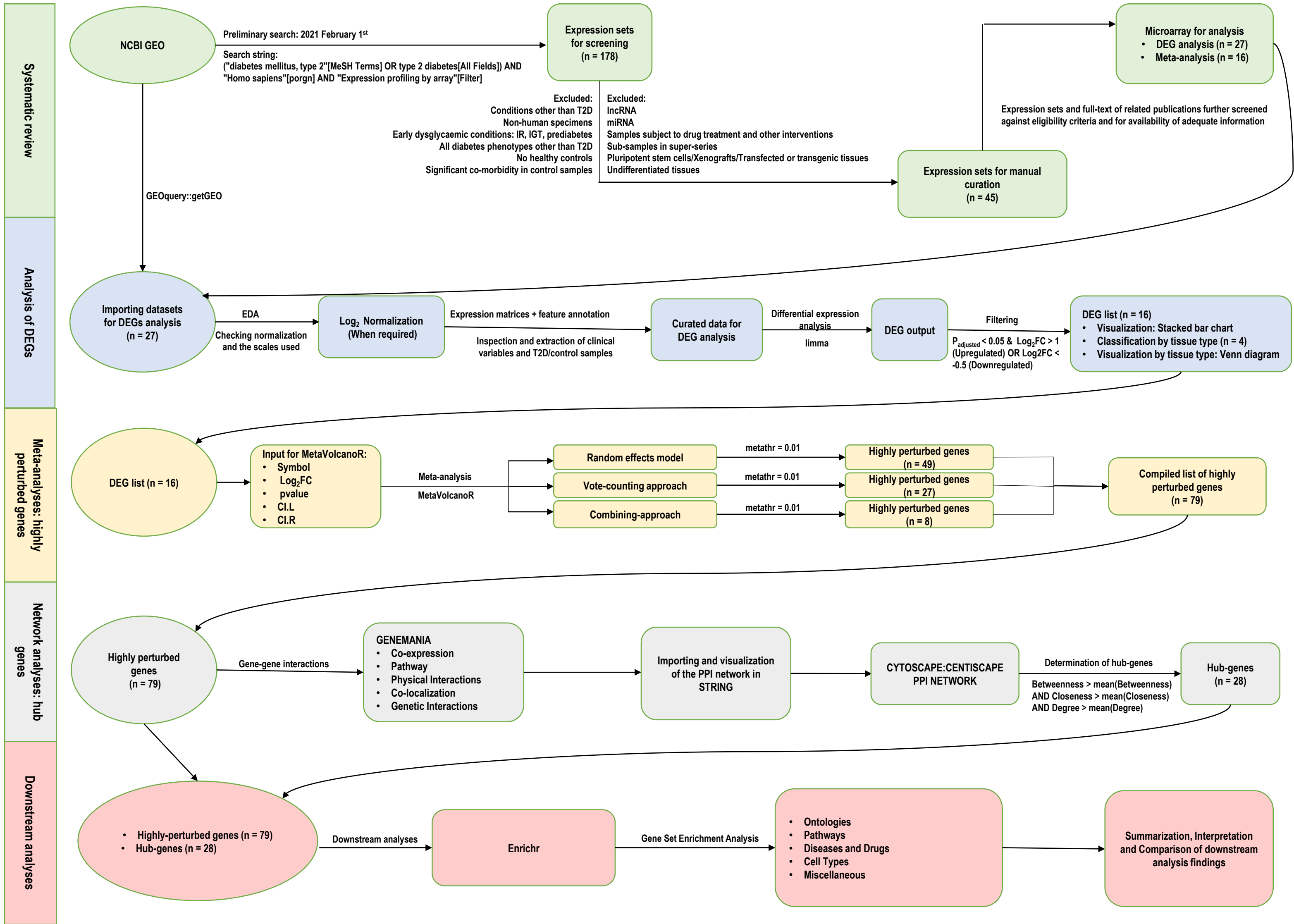

Supplement: Supplementary file 1 — Supplementary file1 (PDF 91 KB) [file 10142_2022_881_MOESM1_ESM.pdf]
